# Parasitic, bacterial, viral, immune-mediated, metabolic and nutritional factors associated with nodding syndrome

**DOI:** 10.1093/braincomms/fcad223

**Published:** 2023-08-17

**Authors:** Arthur W D Edridge, Gasim Abd-Elfarag, Martin Deijs, Melissa H Broeks, Cosimo Cristella, Brandon Sie, Frédéric M Vaz, Judith J M Jans, Job Calis, Hans Verhoef, Ayse Demir, Sven Poppert, Beatrice Nickel, Alje van Dam, Boy Sebit, Maarten J Titulaer, Jaco J Verweij, Menno D de Jong, Tom van Gool, Brian Faragher, Nanda M Verhoeven-Duif, Stephen J Elledge, Lia van der Hoek, Michael Boele van Hensbroek

**Affiliations:** Amsterdam Centre for Global Child Health, Emma Children’s Hospital, Amsterdam UMC, Location University of Amsterdam, 1105 AZ Amsterdam, The Netherlands; Department of Medical Microbiology and Infection Prevention, Amsterdam UMC, Location University of Amsterdam, 1105 AZ Amsterdam, The Netherlands; Amsterdam Centre for Global Child Health, Emma Children’s Hospital, Amsterdam UMC, Location University of Amsterdam, 1105 AZ Amsterdam, The Netherlands; Department of Neurology & Psychiatry, College of Medicine, University of Juba, P.O. Box 82, Juba, South Sudan; Department of Medical Microbiology and Infection Prevention, Amsterdam UMC, Location University of Amsterdam, 1105 AZ Amsterdam, The Netherlands; Department of Genetics, Section Metabolic Diagnostics, University Medical Center Utrecht, 3584 CX Utrecht, The Netherlands; Department of Medical Microbiology and Infection Prevention, Amsterdam UMC, Location University of Amsterdam, 1105 AZ Amsterdam, The Netherlands; Division of Genetics, Brigham and Women’s Hospital, Howard Hughes Medical Institute, Boston, MA 02115, USA; Department of Genetics, Harvard Medical School, Boston, MA 02115, USA; Department of Clinical Chemistry, Amsterdam UMC, Location University of Amsterdam, 1105 AZ Amsterdam, The Netherlands; Department of Genetics, Section Metabolic Diagnostics, University Medical Center Utrecht, 3584 CX Utrecht, The Netherlands; Amsterdam Centre for Global Child Health, Emma Children’s Hospital, Amsterdam UMC, Location University of Amsterdam, 1105 AZ Amsterdam, The Netherlands; Department of Paediatrics and Child Health, Kamuzu University of Health Sciences, P.O. Box 95, Blantyre, Malawi; Division of Human Nutrition and Health, Wageningen University, 6701 AR Wageningen, The Netherlands; Laboratory for Clinical Chemistry and Hematology, Meander Medical Centre, 3813 TZ Amersfoort, The Netherlands; Diagnostic Centre, Swiss Tropical and Public Health Institute, University of Basel, 4123 Allschwil, Switzerland; University of Basel, 4056 Basel, Switzerland; Diagnostic Centre, Swiss Tropical and Public Health Institute, University of Basel, 4123 Allschwil, Switzerland; University of Basel, 4056 Basel, Switzerland; Department of Medical Microbiology and Infection Prevention, Amsterdam UMC, Location University of Amsterdam, 1105 AZ Amsterdam, The Netherlands; Department of Neurology & Psychiatry, College of Medicine, University of Juba, P.O. Box 82, Juba, South Sudan; Department of Neurology, Erasmus MC University Medical Center, 3000 CA Rotterdam, The Netherlands; Microvida Laboratory for Medical Microbiology and Immunology, Elisabeth-Tweesteden Hospital, 5022 GC Tilburg, The Netherlands; Department of Medical Microbiology and Infection Prevention, Amsterdam UMC, Location University of Amsterdam, 1105 AZ Amsterdam, The Netherlands; Department of Medical Microbiology and Infection Prevention, Amsterdam UMC, Location University of Amsterdam, 1105 AZ Amsterdam, The Netherlands; Department of Clinical Sciences, Liverpool School of Tropical Medicine, Liverpool L3 5QA, UK; Department of Genetics, Section Metabolic Diagnostics, University Medical Center Utrecht, 3584 CX Utrecht, The Netherlands; Division of Genetics, Brigham and Women’s Hospital, Howard Hughes Medical Institute, Boston, MA 02115, USA; Department of Medical Microbiology and Infection Prevention, Amsterdam UMC, Location University of Amsterdam, 1105 AZ Amsterdam, The Netherlands; Amsterdam Centre for Global Child Health, Emma Children’s Hospital, Amsterdam UMC, Location University of Amsterdam, 1105 AZ Amsterdam, The Netherlands

**Keywords:** nodding syndrome, *Onchocerca volvulus*, *Mansonella perstans*, encephalopathy

## Abstract

Nodding syndrome is a neglected, disabling and potentially fatal epileptic disorder of unknown aetiology affecting thousands of individuals mostly confined to Eastern sub-Saharan Africa. Previous studies have identified multiple associations—including *Onchocerca volvulus*, antileiomodin-1 antibodies, vitamin B_6_ deficiency and measles virus infection—yet, none is proven causal. We conducted a case-control study of children with early-stage nodding syndrome (symptom onset <1 year). Cases and controls were identified through a household survey in the Greater Mundri area in South Sudan. A wide range of parasitic, bacterial, viral, immune-mediated, metabolic and nutritional risk factors was investigated using conventional and state-of-the-art untargeted assays. Associations were examined by multiple logistic regression analysis, and a hypothetical causal model was constructed using structural equation modelling. Of 607 children with nodding syndrome, 72 with early-stage disease were included as cases and matched to 65 household- and 44 community controls. *Mansonella perstans* infection (odds ratio 7.04, 95% confidence interval 2.28–21.7), *Necator americanus* infection (odds ratio 2.33, 95% confidence interval 1.02–5.3), higher antimalarial seroreactivity (odds ratio 1.75, 95% confidence interval 1.20–2.57), higher vitamin E concentration (odds ratio 1.53 per standard deviation increase, 95% confidence interval 1.07–2.19) and lower vitamin B_12_ concentration (odds ratio 0.56 per standard deviation increase, 95% confidence interval 0.36–0.87) were associated with higher odds of nodding syndrome. In a structural equation model, we hypothesized that *Mansonella perstans* infection, higher vitamin E concentration and fewer viral exposures increased the risk of nodding syndrome while lower vitamin B_12_ concentration, *Necator americanus* and malaria infections resulted from having nodding syndrome. We found no evidence that *Onchocerca volvulus,* antileiomodin-1 antibodies, vitamin B_6_ and other factors were associated with nodding syndrome. Our results argue against several previous causal hypotheses including *Onchocerca volvulus*. Instead, nodding syndrome may be caused by a complex interplay between multiple pathogens and nutrient levels. Further studies need to confirm these associations and determine the direction of effect.

## Teaser

Nodding syndrome is an unexplained encephalopathy affecting thousands of children in sub-Saharan Africa. In a case-control study from South-Sudan, Edridge *et al.* found a complex interplay between infections and nutrient levels but no proof of causation by *O. volvulus* infection, autoimmunity to Leiomodin-1 nor vitamin B_6_ deficiency.

## Graphical Abstract

**Graphical abstract:**
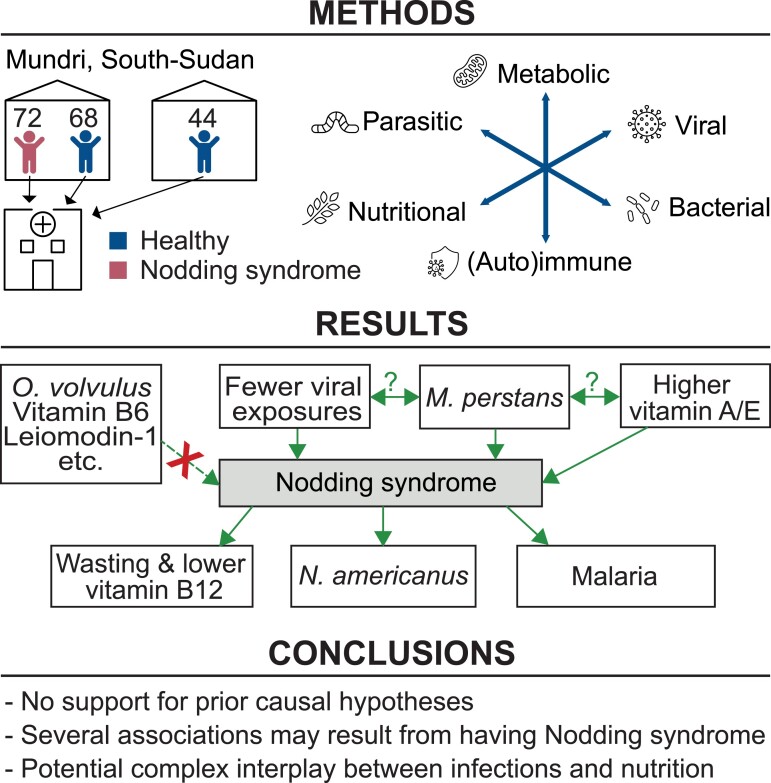

See the scientific commentary for this article ‘New clues to the elusive aetiology of nodding syndrome’, by Peter S. Spencer, https://doi.org/10.1093/braincomms/fcad236.

## Introduction

Nodding syndrome (NS) is a neglected and debilitating neurological disorder of unknown aetiology affecting thousands of children and young adults in sub-Saharan Africa.^1–3^ In high prevalence areas, one in five households can be affected.^4^ Clinical features of NS may include atonic seizures with head nodding, other seizures types, cognitive deficits and stunted growth, and parkinsonism in advanced stages.^2^ Patients are often stigmatized, isolated and sexually abused. While several factors may slow disease progression (e.g. nutritionally balanced diet and anticonvulsive therapy), no curative options are available, and severely diseased individuals often die prematurely.^1–3^

NS has previously been associated with *Onchocerca volvulus* infection,^5–9^ history of measles^10,11^ and nutritional deficiencies (specifically vitamin B_6_ deficiency),^8,12–14^ which resulted in potentially premature public health interventions as proof of causality is lacking.^2,10,15^ Part of the failure to identify the aetiology may be explained by the fact that previous studies were limited in sample size, diagnostic breadth and investigations during late-stage disease when a causative factor may no longer be detectable. We investigated a broad range of possible parasitic, viral, immune-mediated, metabolic and nutritional causes using conventional and state-of-the-art untargeted laboratory methods in children with early-stage NS.

## Materials and methods

Details and references for several laboratory studies can be found in Supplementary Methods.

### Study setting and design

This study was conducted in the Greater Mundri area, Western Equatoria state of South Sudan, with one of the highest estimated NS prevalences.^5,16^ NS-affected children (cases) were identified through a household survey. Individuals meeting all of the following criteria were asked to participate: probable or confirmed NS case definition (Table 1); aged 3–18 years; onset of NS symptoms <1 year ago; no history of epilepsy (other than NS). Each case was age-matched (±3 years) to a healthy household control and a community control (living in the same village, excluding direct neighbours) to control for family-associated and environmental influences. Cases and controls were enrolled simultaneously. Controls with neurological deficits on physical examination, history of coma or repeated convulsions or significant head trauma were excluded.

**Table 1:** Case definition of NS used in this study, adapted from Idro *et al*.^22^

Diagnosis of NS	Criteria
**Probable**	Suspected case, with ‘head nodding’ (as major) and at least 1 minor criterion or with ‘repeated convulsions’ (as major) and at least 2 minor criteria.
	Major criteria:
	Head nodding with a frequency of 5–20 times/min
	History of repeated generalized convulsions
	Minor criteria:
	Other neurologic abnormalities (cognitive decline, school dropout due to cognitive/behavioural problems, other seizures, or neurological abnormalities)
	Nodding or other seizures triggered by food and/or cold weather
	Stunting or wasting
	Clustering in time or space with cases with nodding seizures
	Delayed sexual or physical development
	Psychiatric manifestations
**Confirmed**	Probable case, with documented head nodding seizures:
	Observed and recorded by a trained healthcare worker
	Videotaped head nodding episode
	Video/EEG/EMG documenting head nodding as atonic

Study subjects were transferred to Lui Hospital for physical (including detailed neurologic) examination^12^ and collection of iliac crest skin snips, blood, urine, stool and cerebrospinal fluid (CSF, cases only). Samples were analysed on site, or temporarily stored at −20°C and later at −80°C until analysis elsewhere.

The study protocol was approved by the ethics committee of the Ministry of Health of the Republic of South Sudan (approval date: December 16, 2016) and University of Antwerp, Belgium (reg.nr: B300201526244). Informed consent was obtained from the guardians of all study participants prior to study enrolment. In addition, verbal assent was obtained from children aged 12 years and above.

### Nutritional studies

Sodium, magnesium, calcium, albumin, C-reactive protein and vitamins A (retinol), B_6_ (all vitamers, also in CSF), B_12_ (cobalamin) and E (dl-α-Tocopherol) concentrations were measured in plasma. CSF from Ugandan children with severe acute encephalopathy (including cerebral malaria, bacterial meningitis and viral encephalitis) were used as controls because no CSF was available from NS controls.

### Metabolic studies

Untargeted metabolomics (plasma and CSF) and lipidomics (plasma) were performed to screen for disturbances in metabolic pathways. CSF from the same subset of Ugandan children and Dutch children without a metabolic disease were included as controls.

### Parasitologic and bacteriologic studies

Skin snip (incubated for 1 h in normal saline) and whole blood samples were examined by microscopy to determine the density of microfilariae. Skin snip, plasma and CSF samples were evaluated by conventional polymerase chain reaction (PCR) for microfilariae using pan-filarial primers and *Wolbachia* filarial endosymbionts (Supplementary Table 1). IgG antibodies targeting *O. volvulus* (OV16), all filaria (pan-filaria), *Echinococcus granulosus*, *Fasciola hepatica*, *Strongyloides*, *Toxocara*, *Trichinella*, *Schistosoma*, *Plasmodium* and *Treponema* were quantified. Stool samples were screened by quantitative PCR (qPCR) for *Ancylostoma duodenale*, *Ascaris lumbricoides*, *Enterobius vermicularis*, *Hymenolepis nana*, *Necator americanus*, *Strongyloides stercoralis*, *Taenia saginata*, *Taenia solium*, *Trichuris trichiura, Schistosoma*, *Cryptosporidium parvum*, *Cyclospora*/*Cystoisospora belli*, *Entamoeba histolytica* and *Giardia lamblia*.

### Virologic studies

CSF and ethylenediaminetetraacetic acid plasma from cases were screened for known and novel viruses using VIDISCA viral metagenomics.^17^ If a virus of interest was detected, its prevalence was further investigated in all study subjects using virus-specific qPCRs. Stool samples were tested for the presence of enterovirus and parechovirus by qPCR.

Serum and CSF samples were subjected to VirScan^18^ viral serological profiling to screen for antibodies targeting nearly all known human viruses (and several bacteria, details in Supplementary Methods).

### Auto-immune studies

Rat-brain slide immunohistochemistry was performed on CSF from cases to screen for known and unknown auto-immune neuronal antibodies.^19,20^ Positive samples were subsequently tested by live rodent hippocampal neuron staining for confirmation.^21^ Serum was also screened for the presence of leiomodin-1 antibodies by western blot to evaluate a previously reported association with the presence of these antibodies.^20^

### Whole blood gene expression profiling

Gene expression profiling was performed on whole blood samples to study the role of systemic host immune response in NS.

### Statistical analysis

The association between exposure variables and NS was explored by both conditional and conventional logistic regression using R (version 4.0.3). All variables with *P*-value <0.05 and those deemed clinically relevant were subsequently combined by multiple logistic regression analysis. A stringent *P*-value cut-off was chosen because of the large number of predictors relative to the sample size. The multiple regression model was generated with and without data imputation (using complete observations only). Missing data were imputed assuming missing data at random (see Supplementary Methods and Supplementary Fig. 1) using MICE R package (version 3.13.0). To explore causal pathways, a structural equation model (SEM) was built using AMOS (version 14).

## Results

Between February 2018 and November 2019, 2263 households were visited in a 20 km radius around Mundri town. Six hundred seven resident children fulfilled the NS definition of whom 114 had early-stage disease and 72 consented to participate in the study as cases. These cases were matched to 65 and 44 household and community controls, respectively. Table 2 summarizes the characteristics of the 181 children included in the analysis. A subanalysis of NS cases according to the original case definition^22^ is available in Supplementary Results. Supplementary Table 2 provides an overview of the number of samples available for each test.

**Table 2:** Socio-demographic data, medical history and clinical characteristics of cases and controls

Characteristics	Cases (*n* = 72)	All controls (*n* = 109)	*P*-value
Male sex	43 (60%)	58 (53%)	0.745^a^
Age in years (median, 1^st^–3^rd^ quartile)	15 (9.75–17)	13 (9–15)	0.026^b^
Moru tribe	72 (100%)	109 (100%)	
**Nodding syndrome criteria**			
Head nodding (<20×/minute)	36 (50%)	0%	
From oral history	31 (43%)	0%	
Observed by a clinician	6 (8%)	0%	
Generalized convulsions	55 (76%)	0%	
Neurologic deficit	22 (31%)	0%	
Seizures triggered by food or cold weather	58 (81%)		
Wasted (weight-for-length Z-score < −2)	19 (26%)	2 (2%)	
Stunted (height-for-age Z-score < −2)	0 (0%)	0%	
Delayed sexual or physical development	1 (1%)	0%	
Psychiatric or behavioural symptoms	11 (15%)	0%	
**Nodding syndrome severity stage^23^**			
1	3/66 (4.5%)		
2	25/66 (38%)		
3	38/66 (58%)		
4–5	0/66 (0%)		
**Anticonvulsant use**	51 (71%)	0%	

a Chi-square test.

b Mann–Whitney *U* test.

### Nutrition

Higher vitamin A concentrations [median 1.10 µmol/l versus 0.89 µmol/l, unadjusted odds ratio (UOR) 1.67 per standard deviation (SD) increase, 95% confidence interval (CI) 1.21–2.37], higher vitamin E concentrations (median 16.72 µmol/l versus 13.94 µmol/l, UOR 1.47 per SD increase, 95% CI 1.07–2.07) and lower vitamin B_12_ concentrations (median 348 pmol/l versus 439 pmol/l, UOR 0.68 per SD increase, 95% CI 0.47–0.95) were associated with increased odds of NS (Table 3 and Supplementary Fig. 2). In CSF, no patterns of vitamin B_6_ vitamer concentrations unique to NS cases compared to unmatched Ugandan controls and Dutch reference values were found (Supplementary Results and Supplementary Fig. 3).

**Table 3:** Exposure variables associated with nodding syndrome

Variable	Median (1^st^–3^rd^ quartile)—No./total no. (%)		Unadjusted odds ratio (95% confidence interval)
	Cases (*n* = 72)	All controls (*n* = 109)	
**Filaria**			
Skin snip count^a^ (range, per filaria)	0 (0–25)	0 (0–15)	1.11 (0.98–1.27)
Blood count^a^ (range, per filaria)	0 (0–5)	0 (0–3)	**1.88** (**1.11–3.17)**
*O. volvulus* skin snips PCR positivity	27/69 (39%)	32/106 (30%)	1.69 (0.82–3.47)
*M. perstans* plasma PCR positivity	18/68 (26%)	6/106 (5.7%)	**12.17** (**2.76–53.77)**
OV16 IgG4 seropositivity	39/63 (62%)	45/104 (43%)	1.94 (0.93–4.05)
Pan-filarial IgG seropositivity^b^	58/70 (83%)	76/106 (72%)	2.18 (0.90–5.30)
**Other parasites**			
Malaria microscopy positivity	23/68 (34%)	39/104 (38%)	0.88 (0.43–1.78)
Malaria seroreactivity^c^ (optical density, per SD)	1.49 (1.23–1.76)	1.34 (1.01–1.59)	**1.88** (**1.24–2.85)**
*E. granulosus* IgG seropositity^b^	30/70 (43%)	57/106 (54%)	0.64 (0.31–1.30)
*Fasciola* IgG seropositivity	11/70 (16%)	11/106 (10%)	1.96 (0.68–5.69)
*Schistosoma* IgG seropositivity^d^	22/70 (31%)	52/106 (49%)	**0.44** (**0.21–0.91)**
*Strongyloides* IgG seropositivity^b^	32/70 (46%)	46/106 (43%)	1.25 (0.63–2.50)
*Giardia* stool qPCR positivity	21/71 (30%)	32/102 (31%)	1.02 (0.52–2.00)
*Cyclo/Cystoispora* stool qPCR positivity	6/71 (8.5%)	2/102 (2.0%)	2.79 (0.54–14.56)
*N. americanus* stool qPCR positivity	26/71 (37%)	20/102 (20%)	**2.62** (**1.16–5.92)**
*H. nana* stool qPCR positivity	1/71 (1.4%)	1/102 (1.0%)	2.00 (0.13–31.98)
*Schistosomiasis* stool qPCR positivity	46/71 (65%)	69/102 (68%)	0.90 (0.42–1.93)
*Trichuris* stool qPCR positivity	2/71 (2.8%)	3/102 (2.9%)	0 (0–Inf)
*Strongyloides* stool qPCR positivity	1/71 (1.4%)	6/102 (5.9%)	0.26 (0.03–2.22)
**Viruses**			
Enterovirus stool qPCR positivity	19/71 (27%)	40/102 (39%)	0.49 (0.23–1.08)
Parechovirus stool qPCR positivity	7/71 (9.9%)	11/102 (11%)	1.04 (0.40–2.72)
Anellovirus blood qPCR^e^			
TTV (copies per reaction, per SD)	37 (4–100)	7 (0–52)	1.12 (0.82–1.53)
TTMDV (copies per reaction, per SD)	49 (2–206)	18 (1–163)	1.26 (0.85–1.85)
TTMV (copies per reaction, per SD)	1 (0–3)	1 (0–4)	1.14 (0.83–1.57)
Seropositivity to viruses^f^ (*N*, per SD)	14 (9–19)	18 (12–24)	**0.35** (**0.17–0.69)**
VirScan measles seropositivity	10/70 (14%)	19/103 (18%)	0.73 (0.29–1.81)
**Nutrient markers** ^g^			
Magnesium (mmol/l, per SD)	0.82 (0.79–0.87)	0.84 (0.81–0.91)	0.76 (0.51–1.12)
Calcium (mmol/l, per SD)	2.09 (2.01–2.15)	2.09 (2.09–2.15)	1.32 (0.84–2.07)
Albumin (g/l, per SD)	37.55 (35.70–39.58)	37.9 (35.7–39.9)	1.04 (0.74–1.45)
Sodium (mmol/l, per SD)	139 (137–140)	139 (137–140)	1.30 (0.86–1.96)
Folate (nmol/l, per SD)	22.9 (16.5–29.3)	24.5 (18.4–32.5)	0.68 (0.44–1.04)
Vitamin A (µmol/l, per SD)	1.10 (0.86–1.30)	0.89 (0.70–1.08)	**2.08** (**1.32–3.28)**
Vitamin B_6_ vitamers:			
PA (nmol/l, per SD)	25.2 (19.0–35.3)	25.9 (19.0–31.6)	0.93 (0.60–1.42)
PLP (nmol/l, per SD)	25.4 (19.2–30.4)	25.4 (18.7–37.2)	0.75 (0.46–1.20)
PL (nmol/l, per SD)	8.8 (7.1–11.4)	9.6 (7.1–13.0)	0.78 (0.50–1.20)
PM (nmol/l, per SD)	0.2 (0.1–0.3)	0.2 (0.1–0.3)	0.88 (0.61–1.27)
PN (nmol/l, per SD)	0.1 (0–0.2)	0.1 (0.1–0.2)	0.66 (0.44–1.00)
Vitamin B_12_ (pmol/l, per SD)	348 (267–521)	439 (312–612)	**0.46** (**0.27–0.79)**
Vitamin E (µmol/l, per SD)	16.72 (13.61–19.92)	13.9 (11.4–16.9)	**1.69** (**1.06–2.70)**
**Auto-immunity**			
LMOD1-IgG seropositivity	37/70 (53%)	29/65 (45%)	1.42 (0.68–2.96)
**Inflammatory markers** ^g^			
AGP (g/l, per SD)	0.8 (0.67–1.06)	0.79 (0.68–0.93)	1.38 (0.95–1.99)
CRP (mg/l, per SD)	1.5 (0.6–3.0)	1.2 (0.5–2.4)	1.16 (0.86–1.58)

Presented results are using conditional logistic regression, results of regular logistic regression were largely similar (Supplementary Table 2). Results for individual control groups in Supplementary Table 3. There were insufficient children infected with or positive for *Cryptosporidium*, *E. histolytica*, *Ascaris*, *Enterobius*, *Taenia Ancylostoma,* Trichinella and Toxocara to allow for meaningful analysis.

TTV, torque teno virus; TTMDV, torque teno midi virus (TTMDV); TTMV, torque teno mini virus (TTMV); PA, pyridoxic acid; PLP, pyridoxal-5-phosphate; PL, pyridoxal; PN, pyridoxamine; PM, pyridoxamine; PN, pyridoxine; LMOD1, leiomodin-1; AGP, alpha-1 acid glycoprotein; CRP, C-reactive protein. Significant associations are shown in bold.

a Number of filaria per high power microscopy field.

b Prone to cross-reactivity.

c Because 99% of subjects were seropositive for malaria, association between NS and seroreactivity (optical density signal) was calculated.

d Seropositivy was considered when antibodies to both soluble egg antigen and adult worm extract were detected.

e Viral loads were compared because nearly all subjects were positive by qPCR.

f Odds ratio calculated as per one viral exposure increase.

g Cut-off positivity values are shown in Supplementary Table 4.

### Metabolism

**Figure 1:**
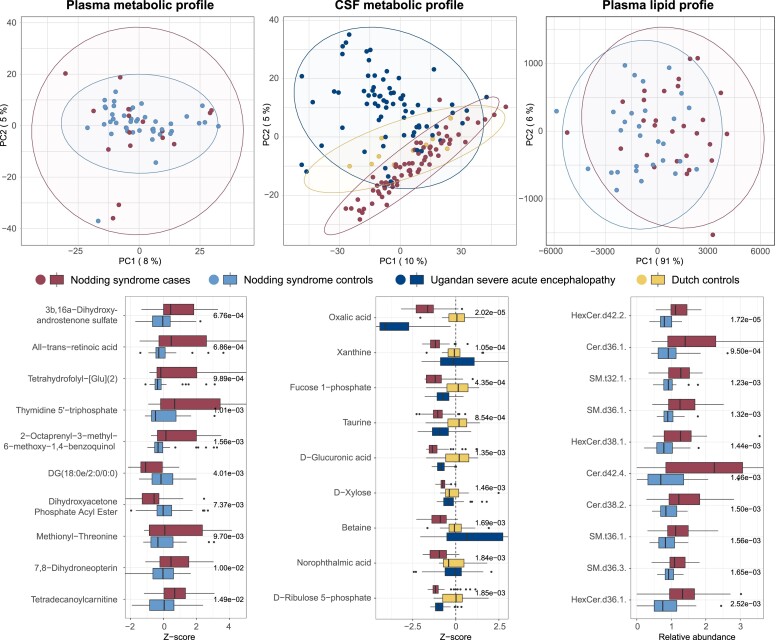
**Metabolomics and lipidomics data on plasma and CSF from nodding syndrome cases and controls.** A total of 1880 unique metabolites in plasma, 1838 unique metabolites in CSF and 1139 unique lipids in plasma were used for analysis. (**Top**) Principal component analysis on results from metabolomics on plasma (left), metabolomics on CSF (middle) and lipidomics on plasma (left) of cases and varying control groups. For the plasma metabolomics analysis, only patients without recent antiepileptic drug use were included, as multiple metabolites were associated with use. Unit variance scaling was applied to the CSF metabolomics data because of the large number of metabolites with extreme values mainly in the Ugandan severe acute encephalopathy group. (**Bottom**) Top 10 features with the highest significance by two sample *t*-test and ANOVA from metabolomics on plasma (left), metabolomics of CSF (right) and lipidomics on plasma (left) between cases and different control groups. As isomers could not be distinguished using Direct-infusion High-resolution Mass Spectrometry, the following isomers correspond to All-*trans*-retinoic acid: 9-*cis*-Retinoic acid; 4-Oxoretinol; 4-OH-Retinal; 9,13-*cis*-Retinoate. For the CSF metabolomics analysis, top features were based on comparisons between cases without antiepileptic drug use and Dutch controls. Unadjusted *P*-values are shown.

Principal component analysis from over 3000 individual metabolites and lipids identified no major differences between the cases and controls (Fig. 1A). The CSF metabolite profile of cases was generally distinct from unmatched Ugandan children with severe acute encephalopathy but comparable to healthy Dutch controls.

On an individual feature level, all-*trans*-retinoic acid (the active metabolite of vitamin A) was among the most significant plasma metabolites (Fig. 1B). However, after correction for multiple testing, no metabolites in plasma, only one plasma lipid [HexCer(d42:2), 1.44-fold higher in cases versus NS controls, adjusted *P* = 0.020], and only one CSF metabolite (oxalic acid, 1.58 Z-score points lower in cases versus Dutch controls, adjusted *P* = 0.038) were significantly different.

### Parasites and bacteria

Higher microfilaria counts in blood (UOR 1.50 per increase in filaria per high power microscopy field, 95% CI 1.02–2.34) and *Mansonella perstans* blood PCR positivity (26% versus 6%, UOR 6.18, 95% CI 2.43–17.9, Table 2, and Supplementary Results) were associated with increased odds of NS. Both (pan-filarial and OV16) serological assays were only associated with NS using conventional but not conditional logistic regression (Supplementary Table 5) and both showed considerable cross-reactivity between filarial species (Supplementary Table 6). None of the CSF samples from cases was positive for filaria or *Wolbachia* filarial endosymbionts by PCR.

Of the non-filarial parasites, infection by *N. americanus* (37% versus 19%, UOR 2.46, 95% CI 1.24–4.92) and *Cyclospora*/*Cystoisospora belli* (9% versus 2%, UOR 4.75, 95% CI 1.06–33.1) were associated with increased odds of NS. Conversely, *Schistosoma* IgG seropositivity was associated with decreased odds of NS (31% versus 49%, UOR 0.48, 95% CI 0.26–0.90), although no association was observed with intestinal *Schistosoma* infection by stool qPCR. Because 99% of the study population was seropositive for malaria, the intensity of seroreactivity (optical density) was evaluated, and stronger seroreactivity was associated with increased odds of NS (median optical density 1.49 AU versus 1.34 AU, UOR 1.6, 95% CI 1.16–2.25). No other parasitic infections were associated with NS. Two of 62 (3%) cases were seropositive for anti-*Treponema* IgG. Because of this low prevalence, it was not further explored in controls.

### Viruses

No evidence of a viral infection was found in CSF by viral metagenomics. In plasma, 38 of 69 (55%) controls contained genomic material from an anellovirus, 16 (23%) from GB virus C, 4 (6%) from hepatitis B virus, 1 (1%) from parvovirus B19, and 1 (1%) from a novel rhabdovirus named Mundri virus.^24^ The viral loads of three anellovirus species were subsequently quantified in cases and controls using qPCR^25^ but were not associated with NS (Table 2). No additional Mundri virus-positive cases or controls were found by qPCR, and serological screening for this virus (to study prior infection) revealed no association with NS.^24^ Hepatitis B virus, parvovirus B19 and GB virus C were not considered to be likely causes of NS and therefore not further investigated.

**Figure 2:**
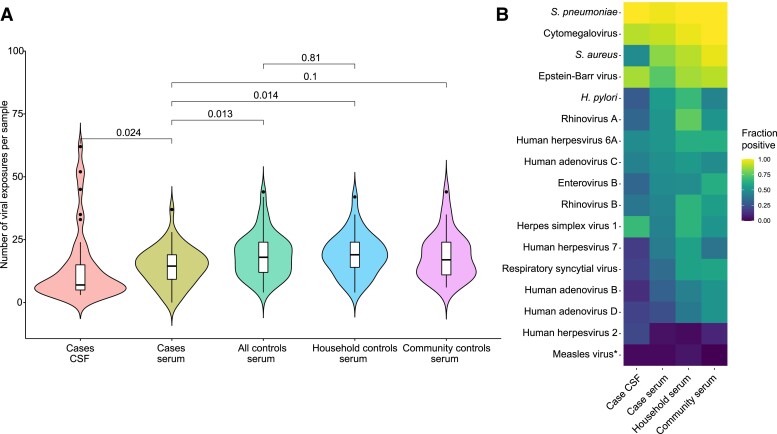
**VirScan viral exposure analysis of nodding syndrome cases and controls.** (**A**) Number of prior viral exposures per sample based on antibody prevalence. The number of viral exposures was counted as described in Materials and methods section. Comparisons between paired groups were calculated by Wilcoxon signed-rank test. (**B**) Seroprevalences of viruses and several bacteria with >50% seroprevalence in cases or one of the control groups. Fisher’s exact test with false discovery rate correction for multiple testing was performed to test for differences, yet no differences were found except for a higher prevalence of antibodies binding *S. aureus* in serum compared to CSF from cases (48% versus 83%, respectively, adjusted *P* = 0.015). *Measles virus was added to the figure despite having a seroprevalence <50% because of a previous association with NS.^10^

Seropositivity to a higher number of viruses by VirScan was associated with lower odds of NS (median 14 in cases versus 18 in all controls, UOR 0.93 per increase of viral exposure, 95% CI 0.88–0.97, Fig. 2A). No association between seropositivity for any specific virus and NS was found, including measles virus (Fig. 2B, additional analyses in Supplementary Results and Supplementary Fig. 4).

### Auto-immunity

Two cases displayed neuropil staining on rat-brain immunohistochemistry using CSF, but none could be confirmed by live hippocampal neuron staining. Seropositivity for leiomodin-1 antibodies was not associated with NS (53% of cases versus 44% of controls, UOR 1.55, 95% CI 0.79–3.04, Table 2). Leiomodin-1 seropositivity was not associated with any filarial assay (Supplementary Table 7).

### Whole blood gene expression

**Figure 3:**
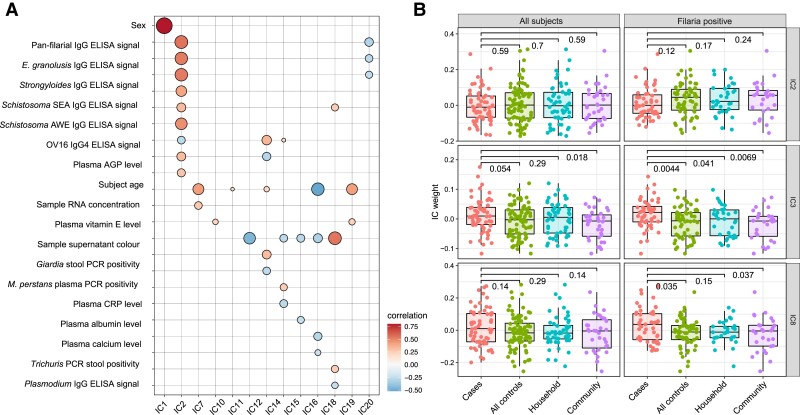
**Gene expression profiling of nodding syndrome cases and controls.** (**A**) Correlation between independent components (ICs) and metadata. The size of the dots and the shade of the colour correspond to the absolute magnitude of the Spearman’s rank correlation coefficient. Only correlations with a Spearman’s rank coefficient >|0.2| (absolute value) and corrected *P*-value <0.05 are shown. (**B**) Comparison of weights (an estimate of the summarized expression of all genes in an IC from a specific subject) of independent components 2, 3 and 8 between cases and controls for all subjects and those seropositive to the pan-filarial assay. Each data point represents one study subject.

Analysis of 11 438 individual gene transcripts did not reveal associations with NS. Twenty independent components (sets of functionally related genes) were identified and several could be associated with clinical and laboratory variables (Fig. 3A). IC2 showed a strong correlation with the pan-filarial serological assay (R = 0.55, *P* < 0.001; Supplementary Table 8). However, none of the independent components could be associated with NS, except for a marginal higher weight of IC3 for cases compared to community controls (Fig. 3B). Subanalysis of filaria-positive patients showed largely similar results. Details in Supplementary Results.

### Multiple logistic regression analysis

**Figure 4:**
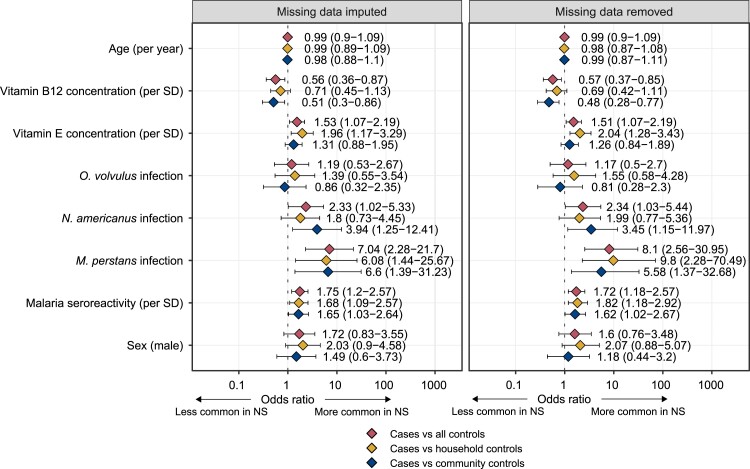
**Multiple logistic regression of factors associated with nodding syndrome.** Vitamin A, folate and number of prior viral exposures were not added because of collinearity with other variables (see Supplementary Table 11 for collinearity analysis of these variables and Supplementary Fig. 8 for a correlation matrix of all independent and dependent variables).

*Mansonella perstans* infection (OR 7.04, 95% CI 2.28–21.7), *N. americanus* infection (OR 2.33, 95% CI 1.02–5.3), antimalarial seroreactivity (OR 1.75 per SD increase, 95% CI 1.20–2.57), vitamin E concentration (OR 1.53 per SD increase, 95% CI 1.07–2.19) and vitamin B_12_ concentration (OR 0.56 per SD increase, 95% CI 0.36–0.87) were associated with NS (Fig. 4).

### Structural equation modelling

**Figure 5:**
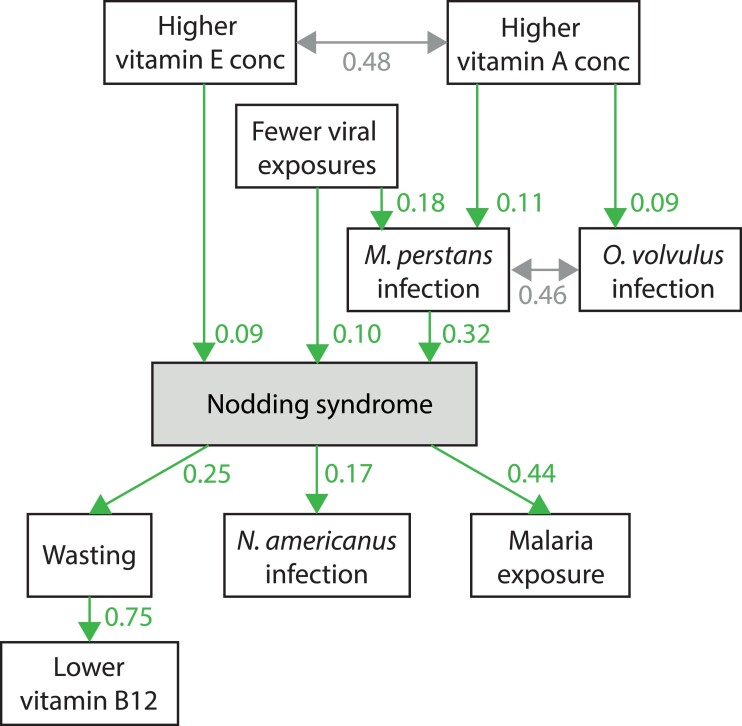
**Simplified structural equation modelling of factors associated with nodding syndrome.** Full model including significance values of all associations in Supplementary Fig. 8. Single headed arrows: positive associations, double headed arrows: covariances, values: unstandardized coefficient estimates that indicate the relative strength of each association. The coefficient estimates are shown as for the absence or presence of dichotomous variables (infections except for malaria and wasting) and per standard deviation for continuous variables (viral exposures, vitamin concentrations and malaria exposure). The root mean square error of approximation, range 0 to 1, a smaller value indicates a better model fit) of the model was 0.041 (90% CI 0.013–0.062).

*Mansonella perstans* infection, higher vitamin E levels and fewer viral exposures were directly associated with increased odds of NS (Fig. 5). Fewer viral exposures had an additional indirect effect by increasing the odds of *M. perstans* infection. Vitamin A levels were only indirectly associated with NS: through an increased risk of *M. perstans* infection and covariance with vitamin E levels. Wasting—which explained the observed differences in vitamin B_12_ levels, *N. americanus* infection and higher antimalarial immunity were directly associated with NS but hypothesized to result from having NS. No direct association between *O. volvulus* and NS could be established.

## Discussion

Multiple infectious, nutritional,\ and immunological factors were associated with NS. Using a SEM, we hypothesize that *M. perstans* infection, higher vitamin A and E concentrations and fewer prior viral exposures directly or indirectly alters the risk of NS, while lower vitamin B_12_ concentration, *N. americanus* infection, and malarial exposure results from having NS. None of the associations were strong enough to suggest a unifactorial cause. Moreover, we did not find proof of association with *O. volvulus*, Leiomodin-1 antibodies, measles virus infection and vitamin B_6_ deficiency, which have previously been suggested as potential causes. Taken together, this suggests that the cause of NS may be more complicated than previously thought, while any definitive proof of causality remains lacking.

The directions of effect in our SEM could be underpinned in several ways. First, as NS patients wander prolonged times outside and sleep isolated from others without bednets,^8,26^ having NS likely increases their exposure to environmental and vector-borne pathogens such as *N. americanus* and malaria. Second, our inclusion of cases with symptoms for less than one year, who were found to be wasted but not stunted—which requires multiple years to occur and is commonly observed in NS patients with a longer presence of disease—^7,8,27^ suggests that malnourishment occurred around or after the onset of NS. This may be explained by the observation that NS patients eat isolated from others and are nutritionally disfavoured during famine.^28^ Moreover, as wasting and vitamin B_12_ levels were strongly correlated, the lower vitamin B_12_ levels likely resulted from having NS. For the other risk factors, we found no convincing arguments to consider them effects of NS. Specifically for *M. perstans*, which is transmitted by biting midges whose bites usually go unnoticed (‘no-see-ums’) and can pass-through bed nets,^29^ the risk of infection may not be affected by having NS. While the SEM showed statistical plausibility, future longitudinal studies are required to confirm the direction of causality.

Despite multiple microscopical, genomic, serological, host immunological and auto-immune assays, we found no evidence of causality for *O. volvulus*, which often is suggested to be the cause of NS.^15^ Combined with previous epidemiological counterarguments (e.g. that *O. volvulus* is endemic in many regions where NS has not been reported),^1,5,30^ one obvious explanation is that onchocerciasis does not cause NS. It may also be possible that we failed to show a true association because of recent ivermectin use which is often given in mass drug administration campaigns to treat onchocerciasis. Yet, one would still expect NS to be associated with OV16 seropositivity which we did not find, as OV16 antigens are produced by adult *O. volvulus* worms that are not killed by ivermectin, and antibodies linger long after microfilarial clearance.^31,32^ Moreover, from the larger epidemiological study in which the current study was embedded, less than half of households used ivermectin in the last five years, and its use was equal between cases and matched household and community controls.^4^ Given the conflicting evidence from our and previous case-control studies, we urge that any interventions to prevent or reduce the NS burden are part of prospective randomized trials which are required to definitively proof or disprove causality.

Instead of *O. volvulus*, we found a strong association between NS and *M. perstans*, as also found by a previous South Sudanese study,^5^ and identified among northern Uganda NS cases.^1^ Generally, mansonellosis causes non-specific symptoms such as fever, fatigue, pruritus, arthralgias and abdominal pain, and only incidentally neurologic symptoms.^33^ Although other studies on NS have not investigated this parasite, previous associations *with O. volvulus* may be reflective of undocumented *M. perstans* infections, as co-infections with *M. perstans* and *O. volvulus* are common,^34^ and filarial serological assays are prone to cross-reactivity.^33^ If *M. perstans* indeed has a causal role in NS, this would have profound consequences for the management of NS. Current public health interventions targeting *O. volvulus* and its vector^35,36^ are already being implemented to reduce the NS burden but are ineffective against *M. perstans.*^37^ Nonetheless, similar to *O. volvulus*, arguments opposing a causal role for *M. perstans* are also present, as it is endemic in many regions where NS has never been documented,^33^ and relatively rare in northern Uganda,^38^ a previous hotspot of incident NS cases.^8,10,39^

Rather than being a cause, filarial infections may also be a proxy marker for a (novel) neurotropic virus that is transmitted by the same vector.^40,41^ To evaluate this hypothesis, as well as that of a post-measles brain disorder,^10^ we performed an exhaustive exploration of a potential viral aetiology through VIDISCA viral metagenomics, specific PCRs, specific serological assays and VirScan viral serological profiling. Although we did find a novel rabies-like virus in one of our NS cases, neither acute or previous infection with this novel virus,^24^ nor with any other virus was associated with NS. Instead, we found that seropositivity to fewer viruses by VirScan was associated with an increased risk of NS. While this may be reflective of differences in vaccination rates, prior viral exposures or immunity, further studies are required to confirm this finding.

Surprisingly, we found that NS cases had higher plasma concentrations of vitamins A and E. This seems not to be the result of vitamin supplementation, since these are nearly exclusively given to malnourished children under 5 years of age, and wasting and vitamins A or E concentrations were not associated in our study. Instead, there was a strong association between higher vitamin A concentration and *M. perstans* infection independent of NS status. Since filaria are known to acquire vitamin A from their hosts and achieve 8-fold higher concentrations than their surrounding host tissues,^42^ the intrafilarial vitamin A may have ‘leaked’ into the plasma after sample collection. Alternatively, it may be possible that vitamin A deficient individuals (present in over 6a0% of our controls) are protected from high microfilaremia, as observed in cotton rats where host vitamin A deficiency impairs the embryogenesis of *Litomosoides carinii* filaria.^43^ We could not find a similar association for vitamin E, yet the correlation between vitamin A and E levels suggests a yet to be explored common causal pathway.

Previous studies also suggested a metabolic aetiology,^44,45^ and specifically, a vitamin B_6_ deficiency.^8,12^ However, using metabolomics, lipidomics and targeted quantification of all vitamin B_6_ vitamers on plasma and CSF, we found no evidence of association. Similarly, certain immune or auto-immune responses have also been associated with NS, specifically auto-immune antibodies targeting Leiomodin-1 resulting from cross-reactivity with *O. volvulus*.^9^ Using whole blood gene expression profiling (studying systemic immune responses), rat-brain slide immunohistochemistry CSF staining (screening for known and novel auto-immune antibodies reacting with brain tissue), and a specific western blot assay for Leiomodin-1 (choice and comparison of assay discussed separately),^20^ we again found no evidence of causality, as also suggested by others.^20,46^

Our study has several limitations. First, age-matching of controls was not feasible for all cases. Second, cases were significantly older than controls (median 15 versus 13 years, respectively), which may especially be relevant in the context of filarial prevalence.^47^ For that reason, we controlled for age in our multiple logistic regression analysis and SEM model. Third, we did not have individual data on medication history such as ivermectin and vitamin use, which would have strengthened our laboratory findings. Fourth, we did not investigate genetic predispositions, which may be required alongside other factors, e.g. filarial infections, to cause NS. Fifth, because it was unethical to obtain CSF from controls, control CSF was used from unmatched Ugandan children. Last, we adapted the original case definition for NS^22^ to also include subjects without nodding seizures but with repeated generalized convulsion and at least two minor criteria. This was done to increase the potential spectrum of clinical presentations associated with NS. To determine whether this may have influenced our results, we performed additional subanalyses using only the ‘nodding’ NS cases but found no major differences (Supplementary Results).

In summary, despite the wide range of causal hypotheses investigated, our results do not demonstrate a definitive cause of NS. Instead, we identified multiple previously known and novel associations and found evidence arguing against several prior causal hypotheses. Given these contrasting findings, we encourage the implementation of potentially preventative or therapeutic interventions, but only as part of clinical trials, as this is the only way of evaluating its effect and is likely required to conclusively proof causation.

## Supplementary Material

fcad223_Supplementary_Data

## Data Availability

Data will be made available for additional analyses upon request.
