# Supplementary material for: Parasitic, bacterial, viral, immune-mediated, metabolic and nutritional factors associated with nodding syndrome
Source: Brain Commun. 2023 Aug 17;5(5):fcad223. doi: 10.1093/braincomms/fcad223 (PMC10507744; doi:10.1093/braincomms/fcad223)
Supplement: fcad223_Supplementary_Data [file fcad223_supplementary_data.zip › Supplementary_Materials.docx]

**SUPPLEMENTARY METHODS**

**Nutritional studies**

Sodium, magnesium, calcium, albumin, and folate concentrations were determined in heparin plasma using the Abbott Architect (Abbott Laboratories) analyzer. C-reactive protein (CRP) and alpha-1-acid glycoprotein (AGP) were included to enable adjustments for differences in micronutrient concentrations caused by acute phase responses. However, as both CRP and AGP were low in nearly all subjects, adjustments were not performed. Vitamins A (retinol), B_12_ (cobalamin), and E (DL-α-Tocopherol) concentrations were determined by ultra-performance liquid chromatography (UPLC, AKSA Medical) in heparin plasma. All vitamin B_6_ vitamers were quantified by UPLC in EDTA plasma and CSF.^1^

**Metabolic studies**

Metabolomics was performed on EDTA plasma (cases and controls) and CSF (cases only) using direct infusion high resolution mass spectrometry (DI-HRMS)^2,3^ Lipidomics was performed on EDTA plasma as previously described.^4^ Results were analyzed by principal component analysis (PCA), two-sample t-test and ANOVA using Metaboanalyst.^5^

**Parasitologic and bacteriologic serological assays**

References to assays used.^6–16^

**VirScan**

Phage immunoprecipitation and sequencing was performed using a phage displayed library of 56-mer linear epitopes from the human virome representing roughly 443 viral species and strains (VirScan) as described previously. VirScan was performed on serum and cerebrospinal fluid of 70 affected individuals, serum of 62 matched sibling healthy controls and serum of 45 community controls. All samples were screened in duplicate. Individual peptide enrichment scores (z-scores) were calculated as described in.^17^ Peptides that produced a z-score greater than 5 in both technical replicates were scored as hits. To make a binary call for whether a particular sample exhibits an IgG response to a particular virus, we followed a method adapted from that described previously.^18^ Briefly, peptides were grouped by viral species of origin, and for each sample the number of peptide hits per virus was counted. Peptide hits that can be explained by cross reactive antibodies were computationally filtered such that peptide hits that share at least a 7 amino acid contiguous sequence identity are only counted once, for the viral species for which the sample had the most peptide hits. Linear regression was then performed to determine the relationship between average number of hits per virus and number of library peptides derived from that virus. We use this regression to set a per-virus threshold for the number of scoring peptides required to indicate a viral exposure.

**Gene expression profiling**

Samples were stabilized in RNAlater, Ambion. RNA was extracted using the RiboPure Blood RNA Purification Kit (Thermo Fischer) and hybridized on a Clariom S Assay HT microarray (Thermo Fischer). Raw array’s intensities were processed using the affy R package (version 1 .74.0). Brainarray custom chip definition file (CDF, version 25) was used when reading in the raw data and downloaded through http://brainarray.mbni.med.umich.edu/Brainarray/Database/CustomCDF/CDF_download.asp. Robust Multi-Average (RMA) normalization was applied to raw data and quality controls were performed by means of ArrayQualityMetrics. Samples that failed to pass the quality control in two or more criteria were excluded from subsequent analyses. Each probeset was annotated using clariomshumanht.db (Brainarray version 25) and converted to official HGNC gene symbols. Only probesets exhibiting a log- expression value greater than 4 in at least 35% of samples were retained. The final gene expression profile included 11,438 genes and 155 samples.

The BIODICA software was used to run independent component analysis (ICA) for deconvolution of gene expression signals. BIODICA implements MATLAB FastICA followed by ICASSO to ensure selection of most stable independent components. All BIODICA’s parameters were left to default and an appropriate number of independent components was determined according to the Keiser’s rule. The goal of ICA is to unmix linearly convoluted patterns of expression, separating them blindly into statistically independent source signals. The two main outputs of ICA are metagenes, representing weighted projections of genes on a given component, and metasamples, representing weighted projections of samples on a given component. The IC expression represents the summarized expression of genes within an IC by a subject as taken from the metasamples.

Signals in the metagenes matrix usually originate from heavy-tailed normal distributions. To identify IC-associated genes, we used false-discovery rate (FDR) estimation by means of the fdrtool R package (version 1.2.17). Genes with a local FDR < 0.001 were deemed significant and included in their respective IC-module. Correlations were calculated between metasamples and metadata (demographics and clinical traits).

R package limma was used to perform differential gene expression analyses. For any gene list presented in the current study, gene set enrichment analysis (GSEA) was performed using the fgsea R package (version 3.14) and comparison gene sets from the molecular signatures database (MSigDB version 7.4).

**Data imputation**

Pattern of missing data were examined prior to data imputation. Of the 181 subjects included, data used for the multiple logistic regression was missing from only 19 (10%) subjects and no specific pattern of missing data was observed (**Supplementary Figure 1**).

**SUPPLEMENTARY RESULTS**

**Filarial PCRs**

The amplicons of pan-filarial PCR positive samples were subsequently sequenced to determine the filarial species. All EDTA plasma samples positive by the pan-filarial PCR (primer DIDR, **Supplementary Table 1**) were classified as *Mansonella perstans* by Sanger sequencing. A random subset of 10 skin snip samples positive by the pan-filarial PCR were analyzed by Sanger sequencing and all characterized as *Onchocerca volvulus*. Nonetheless, a relatively high number of contaminating bands (classified as host genomic contamination) were often present which did not allow fainter bands to be evaluated. Consequently, an alternative PCR with a pan-filarial primary PCR (ITS), followed by a species-specific nested PCR (ITS-Loa for *Loa loa*, and ITS-OV for *O. volvulus*) was used on all skin snip samples (**Supplementary Table 1** for primer details). None of the samples were positive for *Loa loa*.

**Vitamin B_6_ vitamers in CSF**

Three patients, all from the Ugandan cohort, had detectable levels of pyridoxine, suggestive of vitamin B_6_ supplementation, which were accordingly removed from the analysis. Comparison of the vitamer concentration between NS cases and Ugandan controls revealed no differences, except for a higher pyridoxamine level (median 0.6 and 0.3 nmol/L in NS cases versus Ugandan controls respectively, p<0.001) and a lower pyridoxal-5’-phosphate level (median 2.7 versus 6.1 nmol/L in NS cases versus Ugandan controls respectively, p=0.049) in NS cases (**Supplementary Figure 2**). However, the pyridoxamine levels of both the NS cases and Ugandan controls were still largely within normal Dutch reference ranges. Likewise, the pyridoxal-5’-phosphate of both the NS cases and Ugandan controls were nearly all below Dutch reference standards. Given that differences in sample handling and storage cannot be excluded and pyridoxal-5’-phosphate in particular is sensitive to poor storage conditions,^19^ the differences were not interpreted as being reflective of NS.

**VirScan**

Differences between CSF and serum were calculated as this may reveal sign of recent neurotropic infection. No significant differences were found in prevalence of specific viruses between serum and CSF of cases (**Figure 2**). However, on individual peptide level, HSV-1 and CMV exhibited a higher magnitude of peptide enrichment score in CSF than serum, whereas a similar pattern was not observed for other pathogens such as enterovirus C and Staphylococcus aureus (**Supplementary Figure 4A**).

Because of the association between NS and anellovirus infections, the VirScan results of these viruses were evaluated in detail. No differences in anellovirus seroprevalence were observed, yet a three-peptide region from TTMV1 was frequently recognized by both cases and controls (**Supplementary Figure 4B**). Overall, the frequency of peptide enrichment of this region was lower in CSF than serum from cases, but of those positive, CSF samples tended to have higher enrichment scores (**Supplementary Figure 4C**).

Likewise, because of prior associations with measles virus infections,^20,21^ anti-measles antibodies were also analyzed in detail. To obtain the most reliable results, we focused on four measles public epitope peptides. Two were previously identified measles public epitopes,^22^ and two which were the most frequently enriched measles peptides for this cohort (Z-score > 2). We call a sample as indicating prior exposure to measles virus if at least one of these four peptides showed significant enrichment by VirScan (Z-score > 3). _­_No association between presence of anti-measles antibodies was found (odds ratio conditional logistic regression in cases versus all controls 0.73, 95% confidence interval 0.29-1.81, **Table 3** and **Supplementary Table 3**) and the seroprevalence in cases and controls (range 14%-22%) was similar to the seroprevalence of healthy control children from a Nodding syndrome case-control study in north central Uganda (range 15-27%).^21^

For the novel rhabdovirus, samples from the index case did not show increased peptide enrichment scores to epitopes from genetically related viruses. evaluated whether any epitopes from genetically related viruses in the VirScan library provided a high peptide enrichment score for the index case, yet this was not the case. Measles virus was also studied in more detail, as previous studies have shown an association with reported history of measles virus infection. VirScan did not detect differences between cases and controls. Although VirScan may not be able to distant previous infection (which may explain the low overall prevalence), recent (<1 year) infections that would coincide with the onset of NS would most likely be detected.

**Gene expression profiling**

Gene epxression profiling of whole blood provided a total of 11,438 transcripts which were available for stastical analysis after quality control. To deconvolute the large number of signals, ICA was performed to identify independent sets of functionally related genes. Twenty independent components (ICs) were identified and several could be associated with clincal and laboratory parameters (**Figure 3A**). IC2 was of particular interest, showing a strong correlation with filarial infection (R = 0.55 with the pan-filarial serologial assay ELISA signal, p<0.001; **Supplementary Table 8**). However, when comparing the weights of all 20 ICs, no patterns unique to NS could be found, except for a marginal higher weight of IC3 for cases compared to community controls (IC weight 0.0098 versus -0.0072, p=0.02, **Figure 3B** and **Supplementary Figure 5**). When limiting the dataset to patients positive for the pan-filarial serological assay – to investigate potential difference in antifilarial immune responses – similar results were found. The minor differences were that the difference in IC3 was more outspoken (IC weights 0.0219 vs -0.0065, p = 0.004), and in addition, a similar pattern was observed for IC8 (IC weight 0.0364 vs -0.0109, p = 0.035). IC3 was associated with coagulation by GSEA (**Supplementary Table 9**) but no specific biological function could be identified for IC8.

On an individual transcript level, no differentially expressed genes (DEGs) were found between cases and controls (controls combined nor as individual control groups). Similarly, no DEGs were identified when comparing a subset of NS cases and controls positive for the pan-filarial serological assay. Multiple DEGs could be found when comparing pan-filarial IgG seropositive and seronegative patients independent of NS status (**Supplementary Figure 6**) which were associated with eosinophilia by GSEA (**Supplementary Table 10**). Nonetheless, expression levels of these genes did not associate with NS status (**Supplementary Figure 7**).

**Subanalysis of cases with and without nodding seizures**

Of the 72 NS cases, 36 (50%) had nodding seizures (history of or observed during observation). No major differences were oberved when comparing the two groups (**Supplementary Table 12**), yet nodding cases had lower vitamin B12 levels than non-nodding cases (median 324 vs 417 respectively, p=0.025), and more filaria in blood as seen by microscopy (median 0 for both, range 0-5 vs 0-3 respectively, p = 0.049).

Next, we determined the risk factors for disease for the nodding and non-nodding cases separately, compared to all controls combined, using conventional logtistic regression (**Supplementary Table 12**). Overall, the associations were of similar direction and magnitude of effect when using nodding and non-nodding cases. As a result, several risk factors just became, or lost significance. Most notably, an association with skin microscopical positivity (OR 1.93, 95% CI 1.25-3.24) and stool enterovirus PCR positivity (OR 0.32, 95% CI 0.11-0.80) was found, while the association with *N. americanus* infection (OR 1.64, 95% CI 0.66-3.91) and malarial seroreactivity (OR 1.38, 95% CI 0.94-2.09) was lost.

In a multiple logistic regression modle, similar directions and magnitudes of effect were observed between the different case groups. For the nodding cases, the associations with vitamin B12 and vitamin E increased in magnitude, while the associations with *N. americanus* and malaria exposure disappeared.

No DEGs were found when comparin either only nodding or non-nodding cases compared to all controls.

**SUPPLEMENTARY TABLES**

**Supplementary Table 1 –** PCR primer combinations used for filarial and *Wolbachia* PCRs.

| **Sample** | **Target** | **Primer name** | **PCR sequence** | **Primer orientation** | **Primer sequence (5’-3’)** | **Reference** |
| --- | --- | --- | --- | --- | --- | --- |
| CSF | *O. volvulus* | O-150 | Primary | Forward | TGTGGAAATTCACCTAAATATG | ^23^ |
|  |  |  |  | Reverse | AATAACTGATGACCTATGACC |  |
|  | *O. volvulus* | 16s | Primary | Forward | AATTACTCCGGAGTTAACAGG | ^24^ |
|  |  |  |  | Reverse | TCTGTCTCACGACGAACTAAAC |  |
|  | *Wolbachia* | Actin | Primary | Forward | GTGCTACGTTGCTTTGGACT |  |
|  |  |  |  | Reverse | GTAATCACTTGGCCATCAGG |  |
|  | *Wolbachia* | FtsZ | Primary | Forward | GCCGATGCGTTTAGACTTGC |  |
|  |  |  |  | Reverse | TATTCGCCGCAGCATCAACT |  |
| EDTA plasma & skin snip | Pan-filaria | DIDR | Primary | Forward | AGTGCGAATTGCAGACGCATTGAG | ^25^ |
|  |  |  |  | Reverse | AGCGGGTAATCACGACTGAGTTGA |  |
| Skin snip^1^ | Pan-filaria | ITS | Primary | Forward | GGTGAACCTGCGGAAGGATC | ^26^ |
|  |  |  |  | Reverse | CTCAATGCGTCTGCAATTCGC |  |
|  | Loa Loa | ITS-Loa | Nested^&^ | Forward | GATGATGATATATGATGAAG |  |
|  |  |  |  | Reverse | TTAAGCTATCGCTTTATCTTC |  |
|  | O. volvulus | ITS-OV | Nested^&^ | Forward | ATATGTAATGATCATTATTAC | ^%^ |
|  |  |  |  | Reverse | ATCAAATACATATAGTTTGTGTG |  |

^1^The DIDR pan-filarial PCR was initially attempted on skin snips but produced high levels of contamination (probably from host genomic material), consequently, two filarial species-specific nested PCRs targeting *Onchocerca volvlulus* and *Loa loa* were used instead. ^&^Nested PCR on the product of the ITS PCR. ^%^In-house primers, created on an *O. volvulus­*-specific sequence within the ITS amplicon.

**Supplementary Table 2** – Sample availability per assay

| **Assay** | **Cases (n=72)** | **Household controls (n=65)** | **Community controls (n=44)** |
| --- | --- | --- | --- |
| Skin snip microscopy | 72 (100%) | 65 (100%) | 40 (91%) |
| Blood microscopy | 72 (100%) | 65 (100%) | 43 (98%) |
| Malaria microscopy | 68 (94%) | 63 (97%) | 41 (93%) |
| Skin snip *O. volvulus* PCR | 69 (96%) | 63 (97%) | 43 (98%) |
| Blood *M. perstans* PCR | 68 (94%) | 64 (98%) | 42 (95%) |
| OV16 serology | 63 (88%) | 63 (97%) | 41 (93%) |
| Treponemal serology | 62 (86%) | NA | NA |
| Serology (all but OV16, *Treponema* and Leiomodin-1) | 70 (97%) | 63 (97%) | 43 (98%) |
| Stool parasite PCRs | 71 (99%) | 63 (97%) | 39 (89%) |
| Nutrition markers (all but vitamin B_6_ vitamers) | 68 (94%) | 62 (95%) | 43 (98%) |
| Vitamin B_6_ vitamers | 67 (93%) | 63 (97%) | 43 (98%) |
| Stool enterovirus/parechovirus PCRs | 71 (99%) | 63 (97%) | 39 (89%) |
| Anellovirus PCRs | 69 (96%) | 62 (95%) | 40 (91%) |
| Leiomodin-1 antibodies | 70 (97%) | 54 (83%) | 11 (25%) |
| Viral metagenomics (plasma) | 69 (96%) | NA | NA |
| Viral metagenomic (CSF) | 67 (93%) | NA | NA |
| VirScan | 46 (64%) | 35 (54%) | 28 (64%) |
| Gene expression profiling | 63 (88%) | 55 (85%) | 37 (84%) |
| Metabolomics (plasma) | 64 (89%) | NA | 44 (100%) |
| Metabolomic (CSF) | 67 (93%)* | NA | NA |
| Lipidomics | 30 (42%) | NA | 30 (68%) |

*Control CSF from 68 Ugandan children with severe encephalopathy and 10 Dutch non-encephalopathic controls was used.

**Supplementary Table 3** – Exposure variables associated with Nodding syndrome.

| **Variable** | **Median (1st-3rd quartile) - No. /total no. (%)** | | | | **Unadjusted odds ratio (95% confidence interval)** | | |
| --- | --- | --- | --- | --- | --- | --- | --- |
|  | **Cases,**  **N = 72** | **All controls,**  **N = 109** | **Household controls, N = 65** | **Community controls,**  **N = 44** | **Cases vs**  **all controls** | **Cases vs**  **household controls** | **Cases vs**  **community controls** |
| **Filaria** |  |  |  |  |  |  |  |
| Skin snip count* (range, per filaria) | 0 (0-25) | 0 (0-15) | 0 (0-15) | 0 (0-10) | 1.11 (0.98-1.27) | 1.18 (0.94-1.48) | 1.08 (0.94-1.24) |
| Blood count* (range, per filaria) | 0 (0-5) | 0 (0-3) | 0 (0-3) | 0 (0-2) | **1.88 (1.11-3.17)** | **2.06 (1.02-4.16)** | 1.62 (0.89-2.92) |
| *O. volvulus* skin snips PCR positivity | 27/69 (39%) | 32 / 106 (30%) | 17/63 (27%) | 15/43 (35%) | 1.69 (0.82-3.47) | 2.14 (0.87-5.26) | 1.44 (0.62-3.38) |
| *M. perstans* plasma PCR positivity | 18/68 (26%) | 6 / 106 (5.7%) | 3/64 (4.7%) | 3/42 (7.1%) | **12.17 (2.76-53.77)** | **15.00 (1.98-113.56)** | **10 (1.28-78.12)** |
| OV16 IgG4 seropositivity | 39/63 (62%) | 45 / 104 (43%) | 28/63 (44%) | 17/41 (41%) | 1.94 (0.93-4.05) | 1.67 (0.73-3.81) | 2.60 (0.93-7.29) |
| Pan-filarial IgG seropositivity^$^ | 58/70 (83%) | 76 / 106 (72%) | 42/63 (67%) | 34/43 (79%) | 2.18 (0.90-5.30) | 2.14 (0.87-5.26) | 3.5 (0.73-16.85) |
| **Other parasites** |  |  |  |  |  |  |  |
| Malaria microscopy positivity | 23/68 (34%) | 39 / 104 (38%) | 22/63 (35%) | 17/41 (41%) | 0.88 (0.43-1.78) | 1.00 (0.48-2.10) | 0.38 (0.10-1.41) |
| Malaria seroreactivity^&^ (OD, per SD) | 1.49 (1.23-1.76) | 1.34 (1.01-1.59) | 1.32 (0.96-1.58) | 1.34 (1.03-1.60) | **1.88 (1.24-2.85)** | **1.92 (1.22-3.02)** | **2.22 (1.19-4.15)** |
| *E. granulosus* IgG seropositity^$^ | 30/70 (43%) | 57 / 106 (54%) | 32/63 (51%) | 25/43 (58%) | 0.64 (0.31-1.30) | 0.57 (0.24-1.36) | 0.73 (0.29-1.81) |
| *Fasciola* IgG seropositivity | 11/70 (16%) | 11 / 106 (10%) | 4/63 (6.3%) | 7/43 (16%) | 1.96 (0.68-5.69) | 3.00 (0.81-11.08) | 1.25 (0.34-4.65) |
| *Schistosoma* IgG seropositivity^@^ | 22/70 (31%) | 52 / 106 (49%) | 26/63 (41%) | 26/43 (60%) | **0.44 (0.21-0.91)** | 0.57 (0.24-1.36) | **0.38 (0.15-0.96)** |
| *Strongyloides* IgG seropositivity^$^ | 32/70 (46%) | 46 / 106 (43%) | 25/63 (40%) | 21/43 (49%) | 1.25 (0.63-2.50) | 1.27 (0.58-2.80) | 1.29 (0.48-3.45) |
| *Giardia* stool qPCR positivity | 21/71 (30%) | 32 / 102 (31%) | 22/63 (35%) | 10/39 (26%) | 1.02 (0.52-2.00) | 0.81 (0.39-1.69) | 1.80 (0.60-5.37) |
| *Cyclo/Cystoispora* stool qPCR positivity | 6/71 (8.5%) | 2 / 102 (2.0%) | 2/63 (3.2%) | 0/39 (0%) | 2.79 (0.54-14.56) | 2.5 (0.49-12.89) | Inf (0-Inf) |
| *N. americanus* stool qPCR positivity | 26/71 (37%) | 20 / 102 (20%) | 14/63 (22%) | 6/39 (15%) | **2.62 (1.16-5.92)** | 2.14 (0.87-5.26) | **5.00 (1.10-22.82)** |
| *H. nana* stool qPCR positivity | 1/71 (1.4%) | 1 / 102 (1.0%) | 0/63 (0%) | 1/39 (2.6%) | 2.00 (0.13-31.98) | Inf (0-Inf) | 1.00 (0.06-15.99) |
| *Schistosomiasis* stool qPCR positivity | 46/71 (65%) | 69 / 102 (68%) | 39/63 (62%) | 30/39 (77%) | 0.90 (0.42-1.93) | 1.18 (0.53-2.64) | 0.44 (0.14-1.44) |
| *Trichuris* stool qPCR positivity | 2/71 (2.8%) | 3 / 102 (2.9%) | 1/63 (1.6%) | 2/39 (5.1%) | 0 (0-Inf) | 0 (0-Inf) | 0 (0-Inf) |
| *Strongyloides* stool qPCR positivity | 1/71 (1.4%) | 6 / 102 (5.9%) | 3/63 (4.8%) | 3/39 (7.7%) | 0.26 (0.03-2.22) | 0.33 (0.03-3.20) | 0 (0-Inf) |
| **Viruses** |  |  |  |  |  |  |  |
| Enterovirus stool qPCR positivity | 19/71 (27%) | 40 / 102 (39%) | 27/63 (43%) | 13/39 (33%) | 0.49 (0.23-1.08) | **0.35 (0.14-0.90)** | 0.63 (0.20-1.91) |
| Parechovirus stool qPCR positivity | 7/71 (9.9%) | 11 / 102 (11%) | 5/63 (7.9%) | 6/39 (15%) | 1.04 (0.40-2.72) | 1.40 (0.44-4.41) | 0.50 (0.13-2.00) |
| Anellovirus blood qPCR^+^ |  |  |  |  |  |  |  |
| -TTV (copies per reaction, per SD) | 37 (4-100) | 7 (0, 52) | 5 (0-32) | 17 (1-75) | 1.12 (0.82-1.53) | 1.10 (0.76-1.58) | 1.29 (0.67-2.48) |
| -TTMDV (copies per reaction, per SD) | 49 (2-206) | 18 (1, 163) | 2 (1-38) | 106 (23-377) | 1.26 (0.85-1.85) | 2.62 (0.60-11.39) | 0.94 (0.56-1.57) |
| -TTMV (copies per reaction, per SD) | 1 (0-3) | 1 (0-4) | 1 (0-3) | 1 (0-6) | 1.14 (0.83-1.57) | 1.61 (0.69-3.78) | 0.91 (0.58-1.43) |
| Seropositivity to viruses^#^ (N, per SD) | 14 (9-19) | 18 (12-24) | 19 (14-24) | 18 (12-24) | **0.35 (0.17-0.69)** | **0.25 (0.09-0.69)** | 0.43 (0.18-1.01) |
| VirScan measles seropositivity | 10/70 (14%) | 19 / 103 (18%) | 13/60 (22%) | 6/43 (14%) | 0.73 (0.29-1.81) | 0.45 (0.16-1.31) | 1.33 (0.30-5.96) |
| **Nutrient markers^*^** |  |  |  |  |  |  |  |
| Magnesium (mmol/L, per SD) | 0.82 (0.79-0.87) | 0.84 (0.81-0.91) | 0.84 (0.81-0.9) | 0.84 (0.81-0.92) | 0.76 (0.51-1.12) | 0.74 (0.48-1.16) | 0.77 (0.48-1.24) |
| Calcium (mmol/L, per SD) | 2.09 (2.01-2.15) | 2.09 (2.09-2.15) | 2.1 (2.05-2.15) | 2.09 (2.03-2.15) | 1.32 (0.84-2.07) | 1.24 (0.76-2.03) | 1.73 (0.75-4.03) |
| Albumin (g/L, per SD) | 37.55 (35.70-39.58) | 37.9 (35.7-39.9) | 37.95 (35.63-40.20) | 37.70 (35.95-39.55) | 1.04 (0.74-1.45) | 0.97 (0.67-1.40) | 1.22 (0.77-1.94) |
| Sodium (mmol/L, per SD) | 139 (137-140) | 139 (137-140) | 139 (137-140) | 138 (137-140) | 1.30 (0.86-1.96) | 1.21 (0.77-1.91) | 1.61 (0.88-2.94) |
| Folate (nmol/L, per SD) | 22.9 (16.5-29.3) | 24.5 (18.4-32.5) | 23.2 (18.0-30.2) | 27.6 (20.9-32.9) | 0.68 (0.44-1.04) | 0.74 (0.47-1.17) | 0.54 (0.29-1.01) |
| Vitamin A (µmol/L, per SD) | 1.10 (0.86-1.30) | 0.89 (0.70-1.08) | 0.89 (0.71-1.08) | 0.91 (0.64-1.10) | **2.08 (1.32-3.28)** | **2.66 (1.4-5.06)** | **1.91 (1.13-3.24)** |
| Vitamin B_6_ vitamers: |  |  |  |  |  |  |  |
| - PA (nmol/L, per SD) | 25.2 (19.0-35.3) | 25.9 (19.0-31.6) | 24.5 (18.7-31.2) | 26.3 (21.7-33.4) | 0.93 (0.60-1.42) | 1.02 (0.59-1.76) | 0.84 (0.53-1.34) |
| - PLP (nmol/L, per SD) | 25.4 (19.2-30.4) | 25.4 (18.7-37.2) | 26.2 (17.7-36.2) | 23.7 (19.4-37.6) | 0.75 (0.46-1.20) | 0.76 (0.44-1.32) | 0.78 (0.41-1.46) |
| - PL (nmol/L, per SD) | 8.8 (7.1-11.4) | 9.6 (7.1-13.0) | 9.7 (7.1-12.3) | 9.2 (7.5-13.6) | 0.78 (0.50-1.20) | 0.72 (0.43-1.21) | 0.83 (0.49-1.39) |
| - PM (nmol/L, per SD) | 0.2 (0.1-0.3) | 0.2 (0.1-0.3) | 0.2 (0.1-0.3) | 0.2 (0.2-0.2) | 0.88 (0.61-1.27) | 0.96 (0.67-1.38) | 0.77 (0.42-1.43) |
| - PN (nmol/L, per SD) | 0.1 (0-0.2) | 0.1 (0.1-0.2) | 0.1 (0.1-0.2) | 0.1 (0.1-0.2) | 0.66 (0.44-1.00) | **0.58 (0.36-0.93)** | 0.95 (0.52-1.75) |
| Vitamin B_12_ (pmol/L, per SD) | 348 (267-521) | 439 (312-612) | 427 (295-599) | 439 (326-674) | **0.46 (0.27-0.79)** | **0.54 (0.31-0.94)** | **0.35 (0.14-0.87)** |
| Vitamin E (µmol/L, per SD) | 16.72 (13.61-19.92) | 13.9 (11.4-16.9) | 13.93 (11.51-16.40) | 14.29 (11.54-18.84) | **1.69 (1.06-2.70)** | **3.34 (1.47-7.61)** | **1.24 (0.73-2.10)** |
| **Autoimmunity** |  |  |  |  |  |  |  |
| LMOD1-IgG seropositivity | 37/70 (53%) | 29/65 (45%) | 23/57 (40%) | 6/12 (50%) | 1.42 (0.68-2.96) | 1.36 (0.63-2.97) | 2.00 (0.37-10.92) |
| **Inflammatory markers^*^** |  |  |  |  |  |  |  |
| AGP (g/L, per SD) | 0.8 (0.67-1.06) | 0.79 (0.68-0.93) | 0.79 (0.65-0.97) | 0.79 (0.72-0.91) | 1.38 (0.95-1.99) | 1.66 (0.99-2.77) | 1.13 (0.74-1.72) |
| CRP (mg/L, per SD) | 1.5 (0.6-3.0) | 1.2 (0.5-2.4) | 1 (0.3-2.6) | 1.5 (0.8-2.4) | 1.16 (0.86-1.58) | 1.15 (0.82-1.62) | 1.07 (0.67-1.71) |

Presented results are using conditional logistic regression, results of regular logistic regression were largely similar (**Supplementary Table 2**). There were insufficient children infected with or positive for *Cryptosporidium*, *E. histolytica*, *Ascaris*, *Enterobius*, *Taenia* *Ancylostoma,* Trichinella and Toxocara to allow for meaningful analysis. TTV: torque teno virus, TTMDV: torque teno midi virus (TTMDV), TTMV: torque teno mini virus (TTMV), PA: pyridoxic acid, PLP: pyridoxal-5-phosphate, PL: pyridoxal, PN: pyridoxamine, PM: pyridoxamine, PN: pyridoxine, LMOD1: leiomodin-1. Significant associations are shown in bold. ^*^Cut-off positivity values are shown in **Supplementary Table 4**. ^#^Odds ratio calculated as per one viral exposure increase. *Number of filaria per high power microscopy field. ^&^Because 99% of subjects were seropositive for malaria, association between NS and seroreactivity (OD signal) was calculated. ^$^Prone to cross-reactivity. ^@^Seropositivy was considered when antibodies to both soluble egg antigen and adult worm extract were detected. ^+^Viral loads were compared because nearly all subjects were positive by qPCR

**Supplementary Table 4 –** Comparison of regular and conditional unadjusted logistic regression of factors associated with NS.

|  | | **Regular unadjusted odds ratio (95% confidence interval)** | | | | | | **Conditional unadjusted odds ratio (95% confidence interval)** | | | | | |
| --- | --- | --- | --- | --- | --- | --- | --- | --- | --- | --- | --- | --- | --- |
|  | | **Cases vs all controls** | | **Cases vs household** | | **Cases vs community** | | **Cases vs all controls** | | **Cases vs household** | | **Cases vs community** | |
| **Filaria** | |  | |  | |  | |  | |  | |  | |
| Skin snip count (range, per filaria) | | 1.08 (0.98-1.21) | | 1.07 (0.96-1.23) | | 1.08 (0.95-1.28) | | 1.11 (0.98-1.27) | | 1.18 (0.94-1.48) | | 1.08 (0.94-1.24) | |
| Blood count (range, per filaria) | | **1.50 (1.02-2.34)** | | 1.38 (0.92-2.27) | | 1.69 (0.99-3.50) | | **1.88 (1.11-3.17)** | | **2.06 (1.02-4.16)** | | 1.62 (0.89-2.92) | |
| *O. volvulus* skin snips PCR positivity | | 1.49 (0.78-2.82) | | 1.74 (0.84-3.68) | | 1.20 (0.55-2.68) | | 1.69 (0.82-3.47) | | 2.14 (0.87-5.26) | | 1.44 (0.62-3.38) | |
| *M. perstans* plasma PCR positivity | | **6.00 (2.36-17.41)** | | **7.32 (2.31-32.53)** | | **4.68 (1.45-21.00)** | | **12.17 (2.76-53.77)** | | **15 (1.98-113.56)** | | **10 (1.28-78.12)** | |
| OV16 IgG4 seropositivity | | **2.13 (1.13-4.08)** | | **2.03 (1.00-4.17)** | | **2.29 (1.04-5.19)** | | 1.94 (0.93-4.05) | | 1.67 (0.73-3.81) | | 2.60 (0.93-7.29) | |
| Pan-filarial IgG seropositivity | | 1.91 (0.92-4.17) | | **2.42 (1.09-5.58)** | | 1.28 (0.48-3.34) | | 2.18 (0.90-5.30) | | 2.14 (0.87-5.26) | | 3.5 (0.73-16.85) | |
| **Other parasites** | |  | |  | |  | |  | |  | |  | |
| Malaria microscopy positivity | | 0.85 (0.45-1.61) | | 0.95 (0.46-1.97) | | 0.72 (0.32-1.61) | | 0.88 (0.43-1.78) | | 1.00 (0.48-2.10) | | 0.38 (0.10-1.41) | |
| Malaria seroreactivity (OD, per SD) | | **1.60 (1.16-2.25)** | | **1.72 (1.20-2.53)** | | **1.43 (0.96-2.17)** | | **1.88 (1.24-2.85)** | | **1.92 (1.22-3.02)** | | **2.22 (1.19-4.15)** | |
| *E. granulosus* IgG seropositity | | 0.64 (0.35-1.18) | | 0.73 (0.36-1.44) | | 0.54 (0.25-1.16) | | 0.64 (0.31-1.30) | | 0.57 (0.24-1.36) | | 0.73 (0.29-1.81) | |
| *Fasciola* IgG seropositivity | | 1.61 (0.65-3.99) | | 2.75 (0.88-10.37) | | 0.96 (0.35-2.81) | | 1.96 (0.68-5.69) | | 3.00 (0.81-11.08) | | 1.25 (0.34-4.65) | |
| *Schistosoma* IgG seropositivity | | **0.48 (0.25-0.90)** | | 0.66 (0.32-1.33) | | **0.30 (0.14-0.66)** | | **0.44 (0.21-0.91)** | | 0.57 (0.24-1.36) | | **0.38 (0.15-0.96)** | |
| *Strongyloides* IgG seropositivity | | 1.10 (0.60-2.02) | | 1.28 (0.64-2.56) | | 0.88 (0.41-1.89) | | 1.25 (0.63-2.50) | | 1.27 (0.58-2.80) | | 1.29 (0.48-3.45) | |
| *Giardia* stool qPCR positivity | | 0.92 (0.47-1.77) | | 0.78 (0.38-1.62) | | 1.22 (0.51-3.03) | | 1.02 (0.52-2.00) | | 0.81 (0.39-1.69) | | 1.80 (0.60-5.37) | |
| *Cyclo/Cystoispora* stool qPCR positivity | | **4.62 (1.03-32.16)** | | 2.82 (0.62-19.72) | | Inf (0-Inf) | | 2.79 (0.54-14.56) | | 2.5 (0.49-12.89) | | Inf (0-Inf) | |
| *N. americanus* stool qPCR positivity | | **2.37 (1.20-4.76)** | | 2.02 (0.95-4.43) | | **3.18 (1.24-9.32)** | | **2.62 (1.16-5.92)** | | 2.14 (0.87-5.26) | | **5.00 (1.10-22.82)** | |
| *H. nana* stool qPCR positivity | | 1.44 (0.06-36.9) | | Inf (0-Inf) | | 0.54 (0.02-13.99) | | 2.00 (0.13-31.98) | | Inf (0-Inf) | | 1.00 (0.06-15.99) | |
| *Schistosomiasis* stool qPCR positivity | | 0.88 (0.46-1.68) | | 1.13 (0.56-2.30) | | 0.55 (0.22-1.31) | | 0.90 (0.42-1.93) | | 1.18 (0.53-2.64) | | 0.44 (0.14-1.44) | |
| *Trichuris* stool qPCR positivity | | 0.96 (0.12-5.92) | | 1.8 (0.17-39.23) | | 0.54 (0.06-4.62) | | 0 (0-Inf) | | 0 (0-Inf) | | 0 (0-Inf) | |
| *Strongyloides* stool qPCR positivity | | 0.23 (0.01-1.38) | | 0.29 (0.01-2.30) | | 0.17 (0.01-1.39) | | 0.26 (0.03-2.22) | | 0.33 (0.03-3.20) | | 0 (0-Inf) | |
| **Viruses** | |  | |  | |  | |  | |  | |  | |
| Enterovirus stool qPCR positivity | | 0.57 (0.29-1.08) | | **0.49 (0.23-1.00)** | | 0.73 (0.31-1.73) | | 0.49 (0.23-1.08) | | **0.35 (0.14-0.9)** | | 0.63 (0.20-1.91) | |
| Parechovirus stool qPCR positivity | | 0.90 (0.32-2.42) | | 1.27 (0.38-4.49) | | 0.60 (0.19-2.01) | | 1.04 (0.40-2.72) | | 1.40 (0.44-4.41) | | 0.50 (0.13-2.00) | |
| Anellovirus blood qPCR | |  | |  | |  | |  | |  | |  | |
| -TTV (*Alphatorquevirus)* | | 1.07 (0.78-1.51) | | 1.1 (0.77-1.78) | | 1.07 (0.65-1.95) | | 1.12 (0.82-1.53) | | 1.10 (0.76-1.58) | | 1.29 (0.67-2.48) | |
| -TTMDV (copies per reaction, per SD) | | 1.21 (0.88-1.86) | | 1.9 (0.98-7.69) | | 1.02 (0.74-1.54) | | 1.26 (0.85-1.85) | | 2.62 (0.6-11.39) | | 0.94 (0.56-1.57) | |
| -TTMV (copies per reaction, per SD) | | 1.13 (0.83-1.64) | | 1.55 (0.91-5.6) | | 0.99 (0.72-1.45) | | 1.14 (0.83-1.57) | | 1.61 (0.69-3.78) | | 0.91 (0.58-1.43) | |
| Seropositivity to viruses (N, per SD) | | **0.53 (0.33-0.82)** | | **0.50 (0.28-0.82)** | | **0.58 (0.33-0.95)** | | **0.35 (0.17-0.69)** | | **0.25 (0.09-0.69)** | | **0.43 (0.18-1.01)** | |
| VirScan measles seropositivity | 0.74 (0.31-1.67) | | 0.6 (0.24-1.49) | | 1.03 (0.35-3.24) | | 0.73 (0.29-1.81) | | 0.45 (0.16-1.31) | | 1.33 (0.30-5.96) | |  |
| **Nutrient markers** | |  | |  | |  | |  | |  | |  | |
| Magnesium (mmol/L, per SD) | | 0.78 (0.56-1.06) | | 0.80 (0.55-1.15) | | 0.71 (0.47-1.06) | | 0.76 (0.51-1.12) | | 0.74 (0.48-1.16) | | 0.77 (0.48-1.24) | |
| Calcium (mmol/L, per SD) | | 1.30 (0.91-2.11) | | 1.27 (0.85-2.13) | | 1.44 (0.89-2.84) | | 1.32 (0.84-2.07) | | 1.24 (0.76-2.03) | | 1.73 (0.75-4.03) | |
| Albumin (g/L, per SD) | | 1.00 (0.74-1.37) | | 0.99 (0.70-1.39) | | 1.03 (0.69-1.52) | | 1.04 (0.74-1.45) | | 0.97 (0.67-1.40) | | 1.22 (0.77-1.94) | |
| Sodium (mmol/L, per SD) | | 1.30 (0.93-1.94) | | 1.22 (0.82-1.91) | | 1.51 (0.98-2.52) | | 1.30 (0.86-1.96) | | 1.21 (0.77-1.91) | | 1.61 (0.88-2.94) | |
| Folate (nmol/L, per SD) | | 0.76 (0.55-1.04) | | 0.86 (0.61-1.20) | | **0.62 (0.40-0.93)** | | 0.68 (0.44-1.04) | | 0.74 (0.47-1.17) | | 0.54 (0.29-1.01) | |
| Vitamin A (µmol/L, per SD) | | **1.67 (1.21-2.37)** | | **1.79 (1.21-2.75)** | | **1.65 (1.10-2.57)** | | **2.08 (1.32-3.28)** | | **2.66 (1.40-5.06)** | | **1.91 (1.13-3.24)** | |
| Vitamin B_6_ vitamers | |  | |  | |  | |  | |  | |  | |
| - PA (nmol/L, per SD) | | 0.96 (0.69-1.30) | | 1.05 (0.71-1.58) | | 0.86 (0.59-1.26) | | 0.93 (0.6-1.42) | | 1.02 (0.59-1.76) | | 0.84 (0.53-1.34) | |
| - PLP (nmol/L, per SD) | | 0.86 (0.59-1.17) | | 0.88 (0.60-1.24) | | 0.83 (0.52-1.27) | | 0.75 (0.46-1.2) | | 0.76 (0.44-1.32) | | 0.78 (0.41-1.46) | |
| - PL (nmol/L, per SD) | | 0.85 (0.61-1.16) | | 0.85 (0.58-1.23) | | 0.82 (0.54-1.23) | | 0.78 (0.50-1.2) | | 0.72 (0.43-1.21) | | 0.83 (0.49-1.39) | |
| - PM (nmol/L, per SD) | | 0.91 (0.62-1.25) | | 0.94 (0.65-1.30) | | 0.81 (0.46-1.40) | | 0.88 (0.61-1.27) | | 0.96 (0.67-1.38) | | 0.77 (0.42-1.43) | |
| - PN (nmol/L, per SD) | | **0.71 (0.49-1.00)** | | **0.64 (0.43-0.91)** | | 0.93 (0.57-1.52) | | **0.66 (0.44-1.00)** | | **0.58 (0.36-0.93)** | | 0.95 (0.52-1.75) | |
| Vitamin B_12_ (pmol/L, per SD) | | **0.68 (0.47-0.95)** | | 0.74 (0.50-1.09) | | **0.62 (0.41-0.91)** | | **0.46 (0.27-0.79)** | | **0.54 (0.31-0.94)** | | **0.35 (0.14-0.87)** | |
| Vitamin E (µmol/L, per SD) | | **1.47 (1.07-2.07)** | | **1.89 (1.25-2.99)** | | **1.20 (0.84-1.75)** | | **1.69 (1.06-2.70)** | | **3.34 (1.47-7.61)** | | **1.24 (0.73-2.10)** | |
| **Autoimmunity** | |  | |  | |  | |  | |  | |  | |
| LMOD1-IgG seropositivity | | 1.39 (0.71-2.75) | | 1.51 (0.74-3.11) | | 0.93 (0.25-3.38) | | 1.42 (0.68-2.96) | | 1.36 (0.63-2.97) | | 2.00 (0.37-10.92) | |
| **Inflammatory markers** | |  | |  | |  | |  | |  | |  | |
| AGP (g/L, per SD) | | 1.22 (0.90-1.66) | | 1.2 (0.85-1.72) | | 1.24 (0.85-1.89) | | 1.38 (0.95-1.99) | | 1.66 (0.99-2.77) | | 1.13 (0.74-1.72) | |
| CRP (mg/L, per SD) | | 1.17 (0.86-1.63) | | 1.15 (0.83-1.73) | | 1.17 (0.81-2.00) | | 1.16 (0.86-1.58) | | 1.15 (0.82-1.62) | | 1.07 (0.67-1.71) | |

**Supplementary Table** 5 - Cut-off values for serological assays.

| **ELISA antigen** | **Positivity cut-off value (optical density)** |
| --- | --- |
| Trichinella | ≥ 0.70 |
| Toxocara | ≥ 0.70 |
| Fasciola | ≥ 0.50 |
| Schistosoma adult worm extract | ≥ 0.30 |
| Schistosoma soluble egg antigen | ≥ 0.60 |
| Pan filaria | ≥ 0.70 |
| Strongyloides | ≥ 0.70 |
| Malaria | ≥ 0.30 |

**Supplementary Table 6** – Association between anti-filarial antibodies and filaria detected in different sample types.

| **Seropositive for** | **Filaria detected in (method)** | **UOR (95% CI)** | **AOR (95% CI)** |
| --- | --- | --- | --- |
| OV16 | Skin snip (microscopy) | **6.59 (2.94-16.4)** | 2.19 (0.71-6.83) |
|  | Skin snip (*O. volvulus* PCR) | **9.16 (4.39-20.5)** | **5.61 (2.05-15.34)** |
|  | Whole blood (microscopy) | **2.68 (1.32-5.66)** | 1.00 (0.4-2.51) |
|  | Plasma (*M. perstans* PCR) | **5.06 (1.77-18.2)** | **5.17 (1.5-17.83)** |
| Pan-filarial antigen | Skin snip (microscopy) | **8.51 (2.45-53.8)** | 2.85 (0.52-15.57) |
|  | Skin snip (*O. volvulus* PCR) | **9.26 (3.16-39.6)** | **4.11 (1.01-16.82)** |
|  | Whole blood (microscopy) | **21.4 (4.43-386)** | **13.27 (1.72-102.4)** |
|  | Plasma (*M. perstans* PCR) | 3.56 (0.98-22.9) | 2.55 (0.52-12.6) |

UOR: unadjusted odds ratio, AOR: adjusted odds ratio. AOR was performed as a logistic regression adjusted for all other filarial detection methods. Significant ORs are in bold.

**Supplementary Table 7** – association between LMOD-1 seropositivity and other filarial assays

| **Characteristic** | **UOR** |
| --- | --- |
| OV16 IgG4 positivity | 1.01 (0.51-2.00) |
| Pan-filarial IgG positivity | 0.70 (0.33-1.48) |
| Blood microscopy positivity | 0.62 (0.28-1.34) |
| Skin microscopy positivity | 0.80 (0.36-1.72) |
| *M. perstans* blood PCR positivity | 1.04 (0.41-2.66) |
| *O. volvulus* skin PCR positivity | 0.70 (0.34-1.43) |

**Supplementary Table 8** – Gene set enrichment analysis of independent component 2 identified by whole blood gene expression profiling of NS cases and controls using the immunologic gene sets from the Molecular Signatures Database (version 7.5.1).

| **Pathway** | **Adjusted p value** | **NES** |
| --- | --- | --- |
| GSE3982_EOSINOPHIL_VS_NKCELL_UP | 4.79E-12 | 2.803205 |
| GSE3982_EOSINOPHIL_VS_DC_UP | 1.13E-11 | 2.786529 |
| GSE3982_EOSINOPHIL_VS_BCELL_UP | 6.70E-11 | 2.795624 |
| GSE3982_EOSINOPHIL_VS_EFF_MEMORY_CD4_TCELL_UP | 2.08E-09 | 2.569037 |
| GSE3982_EOSINOPHIL_VS_CENT_MEMORY_CD4_TCELL_UP | 2.27E-09 | 2.57179 |
| GSE3982_BASOPHIL_VS_CENT_MEMORY_CD4_TCELL_UP | 6.77E-08 | 2.474485 |
| GSE37416_CTRL_VS_24H_F_TULARENSIS_LVS_NEUTROPHIL_UP | 4.16E-07 | 2.455511 |
| GSE36392_TYPE_2_MYELOID_VS_NEUTROPHIL_IL25_TREATED_LUNG_UP | 4.27E-07 | 2.523636 |
| GSE3982_EOSINOPHIL_VS_TH2_UP | 1.19E-06 | 2.481136 |
| GSE3982_EOSINOPHIL_VS_MAST_CELL_UP | 1.69E-05 | 2.389681 |

NES: normalized enrichment score. Top 20 gene sets by significance are shown.

**Supplementary Table 9** – Gene set enrichment analysis of independent component 3 identified by whole blood gene expression profiling of NS cases and controls using the GO gene sets from the Molecular Signatures Database (version 7.5.1).

| **Pathway** | **Adjusted p value** | **NES** |
| --- | --- | --- |
| GOBP_WOUND_HEALING | 6.51E-17 | 2.636376 |
| GOBP_RESPONSE_TO_WOUNDING | 4.96E-15 | 2.462905 |
| GOBP_HEMOSTASIS | 1.35E-14 | 2.845549 |
| GOBP_PLATELET_ACTIVATION | 1.60E-11 | 2.94684 |
| GOBP_REGULATION_OF_BODY_FLUID_LEVELS | 3.29E-11 | 2.489936 |
| GOBP_PLATELET_AGGREGATION | 2.15E-07 | 3.069491 |
| GOBP_HOMOTYPIC_CELL_CELL_ADHESION | 5.42E-07 | 2.932559 |
| GOBP_REGULATION_OF_WOUND_HEALING | 1.24E-06 | 2.908424 |
| GOBP_NEGATIVE_REGULATION_OF_WOUND_HEALING | 2.85E-06 | 3.124171 |
| GOBP_REGULATION_OF_RESPONSE_TO_WOUNDING | 4.88E-06 | 2.686337 |

**Supplementary Table 10** – Gene set enrichment analysis of genes identified by whole blood gene expression profiling of pan-filarial seropositive versus seronegative patients using the immunologic gene sets from the Molecular Signatures Database (version 7.5.1).

| **Pathway** | **Adjusted p value** | **NES** |
| --- | --- | --- |
| GSE3982_EOSINOPHIL_VS_CENT_MEMORY_CD4_TCELL_UP | 9.30E-13 | -2.57262 |
| GSE22886_NAIVE_BCELL_VS_NEUTROPHIL_DN | 1.07E-10 | -2.41984 |
| GSE3982_NEUTROPHIL_VS_NKCELL_UP | 1.07E-10 | -2.43858 |
| GSE34205_RSV_VS_FLU_INF_INFANT_PBMC_UP | 5.32E-10 | 2.563039 |
| GSE10325_LUPUS_CD4_TCELL_VS_LUPUS_BCELL_DN | 6.55E-10 | -2.38302 |
| GSE3982_EOSINOPHIL_VS_EFF_MEMORY_CD4_TCELL_UP | 8.54E-10 | -2.34413 |
| GSE3982_EOSINOPHIL_VS_NKCELL_UP | 9.56E-09 | -2.36369 |
| GSE3982_NEUTROPHIL_VS_EFF_MEMORY_CD4_TCELL_UP | 3.99E-08 | -2.22358 |
| GSE29618_BCELL_VS_PDC_UP | 8.24E-08 | -2.19289 |
| GSE33425_CD8_ALPHAALPHA_VS_ALPHABETA_CD161_HIGH_TCELL_UP | 1.14E-07 | -2.2049 |

**Supplementary Table 11** – Evaluation of collinearity between independent variables.

|  | Effect on each other | | Effect on NS | |
| --- | --- | --- | --- | --- |
|  | Pearson’s correlation estimate (95% CI) | Adjusted odds ratio^$^  (95% CI) | One included^%^ | Both included^%^ |
| Pairs of independent variables |  |  | Odds ratio (95% CI) | Odds ratio (95% CI) |
| Vitamin E (per SD)* | 0.49 (0.36-0.59) | 1.60 (1.40-1.83) | 1.55 (1.09-2.21) | 1.46 (0.98-2.17) |
| Vitamin A (per SD) |  |  | 1.43 (0.96-2.13) | 1.17 (0.74-1.85) |
| Vitamin B_12_ (per SD)* | 0.18 (0.04-0.33) | 1.18 (1.02-1.37) | 0.59 (0.39-0.89) | 0.62 (0.41-0.93) |
| Folic acid (per SD) |  |  | 0.68 (0.45-1.01) | 0.73 (0.49-1.09) |
| *M. perstans* PCR positivity | -0.30 (-0.46- -0.12) | 0.71 (0.53-0.94) | 6.36 (1.99-20.3) | 3.96 (1.03-15.31) |
| Prior viral exposures (per SD)* |  |  | 0.26 (0.06-1.16) | 0.33 (0.07-1.63) |
| *M. perstans* PCR positivity | 0.33 (0.19-0.46) | 2.56 (1.45-3.50) | 6.36 (1.99-20.3) | 3.33 (0.8-13.87) |
| Vitamin A (per SD)* |  |  | 1.48 (0.89-2.45) | 1.3 (0.77-2.21) |

*****Variable used as independent variable in the regression analysis between paired independent variables. ^&^The adjusted odds ratios between the paired independent variables by regression analysis corrected for NS status. ^%^Odds ratio of effect on NS estimated by multiple logistic regression using data imputation including all variables as **Figure 4** except for the paired independent variable considered here.

**Supplementary Table 12** – Differences between nodding and non-nodding cases

|  |  |  | **Median (IQR); N (%)** | |  | **Regular logistic regression OR (95% CI) vs all controls** | | |
| --- | --- | --- | --- | --- | --- | --- | --- | --- |
|  |  |  | **Nodding cases** | **Non-nodding cases** | **p-value** | **All cases** | **Nodding cases** | **Non-nodding cases** |
| **Filaria** |  |  |  |  |  |  |  |  |
| Microscopical skin snip count | | | 0 (0, 5) | 0 (0, 0) | 0.083 | 1.08 (0.98-1.21) | **1.15 (1.04-1.31)** | 0.86 (0.61-1.07) |
| Microscopical blood count | | | 0 (0, 1) | 0 (0, 0) | **0.049** | **1.50 (1.02-2.34)** | **1.93 (1.25-3.24)** | 0.91 (0.41-1.78) |
| *O. volvulus* skin snips PCR positivity | | | 17 (49%) | 10 (29%) | 0.1 | 1.49 (0.78-2.82) | 2.18 (1.00-4.80) | 0.96 (0.40-2.20) |
| *M. perstans* plasma PCR positivity | | | 8 (24%) | 10 (29%) | 0.6 | **6.00 (2.36-17.41)** | **5.13 (1.64-16.85)** | **6.94 (2.35-22.23)** |
| OV16 IgG4 seropositivity | |  | 23 (66%) | 16 (57%) | 0.5 | **2.13 (1.13-4.08)** | **2.51 (1.15-5.73)** | 1.75 (0.76-4.13) |
| Pan-filarial IgG seropositivity | | | 29 (83%) | 29 (83%) | >0.9 | 1.91 (0.92-4.17) | 1.91 (0.76-5.50) | 1.91 (0.76-5.50) |
| **Other parasites** | |  |  |  |  |  |  |  |
| Malaria microscopy positivity | | | 10 (29%) | 13 (39%) | 0.3 | 0.85 (0.45-1.61) | 0.67 (0.28-1.5) | 1.08 (0.48-2.40) |
| Malaria seroreactivity (OD) | | | 1.43 (1.18, 1.75) | 1.55 (1.30, 1.76) | 0.4 | **1.60 (1.16-2.25)** | 1.38 (0.94-2.09) | **1.91 (1.24-3.11)** |
| *E. granulosus* IgG seropositity | | | 17 (49%) | 13 (37%) | 0.3 | 0.64 (0.35-1.18) | 0.81 (0.38-1.75) | 0.51 (0.23-1.10) |
| *Fasciola* IgG seropositivity | |  | 7 (20%) | 4 (11%) | 0.3 | 1.61 (0.65-3.99) | 2.16 (0.73-6.02) | 1.11 (0.29-3.53) |
| *Schistosoma* IgG seropositivity | | | 14 (39%) | 8 (22%) | 0.12 | **0.48 (0.25-0.90)** | 0.70 (0.32-1.49) | **0.31 (0.12-0.72)** |
| *Strongyloides* IgG seropositivity | | | 17 (49%) | 15 (43%) | 0.6 | 1.10 (0.60-2.02) | 1.23 (0.57-2.66) | 0.98 (0.45-2.11) |
| *Giardia* stool qPCR positivity | | | 8 (23%) | 13 (36%) | 0.2 | 0.92 (0.47-1.77) | 0.65 (0.25-1.53) | 1.24 (0.55-2.73) |
| *Cyclo/Cystoispora* stool qPCR positivity | | | 4 (11%) | 2 (5.6%) | 0.4 | **4.62 (1.03-32.16)** | **6.45 (1.20-48.16)** | 2.94 (0.34-25.29) |
| *N. americanus* stool qPCR positivity | | | 10 (29%) | 16 (44%) | 0.2 | **2.37 (1.20-4.76)** | 1.64 (0.66-3.91) | **3.28 (1.44-7.5)** |
| *H. nana* stool qPCR positivity | | | 0 (0%) | 1 (2.8%) | >0.9 | 1.44 (0.06-36.9) | NA | 2.89 (0.11-74.28) |
| *Schistosomiasis* stool qPCR positivity | | | 26 (74%) | 20 (56%) | 0.1 | 0.88 (0.46-1.68) | 1.38 (0.60-3.42) | 0.60 (0.27-1.31) |
| *Trichuris* stool qPCR positivity | | | 0 (0%) | 2 (5.6%) | 0.5 | 0.96 (0.12-5.92) | NA | 1.94 (0.25-12.19) |
| *Strongyloides* stool qPCR positivity | | | 1 (2.9%) | 0 (0%) | 0.5 | 0.23 (0.01-1.38) | 0.47 (0.02-2.89) | NA |
| **Viruses** |  |  |  |  |  |  |  |  |
| Enterovirus stool qPCR positivity | | | 6 (17%) | 13 (36%) | 0.071 | 0.57 (0.29-1.08) | **0.32 (0.11-0.8)** | 0.88 (0.39-1.91) |
| Parechovirus stool qPCR positivity | | | 5 (14%) | 2 (5.6%) | 0.3 | 0.90 (0.32-2.42) | 1.38 (0.41-4.13) | 0.49 (0.07-1.93) |
| Anellovirus blood qPCR^+^ | |  |  |  |  |  |  |  |
| -TTV (copies per reaction) | | | 44 (8, 150) | 16 (3, 66) | 0.2 | 1.07 (0.78-1.51) | 1.13 (0.79-1.61) | 0.98 (0.56-1.38) |
| -TTMDV (copies per reaction) | | | 87 (16, 209) | 24 (1, 140) | 0.12 | 1.21 (0.88-1.86) | 0.72 (0.15-1.61) | 1.35 (0.98-2.14) |
| -TTMV (copies per reaction) | | | 1 (0, 4) | 1 (0, 2) | 0.2 | 1.13 (0.83-1.64) | 0.39 (0.01-1.29) | 1.27 (0.93-1.88) |
| Seropositivity to viruses | | | 15 (14, 18) | 12 (8, 20) | 0.4 | **0.53 (0.33-0.82)** | 0.55 (0.24-1.09) | **0.53 (0.32-0.84)** |
| VirScan measles seropositivity | | | 5 (14%) | 5 (14%) | >0.9 | 0.74 (0.31-1.67) | 0.74 (0.23-2.02) | 0.74 (0.23-2.02) |
| **Nutrient markers** | |  |  |  |  |  |  |  |
| Magnesium (mmol/L) | | | 0.84 (0.80, 0.86) | 0.82 (0.79, 0.88) | >0.9 | 0.78 (0.56-1.06) | 0.78 (0.53-1.14) | 0.80 (0.53-1.17) |
| Calcium (mmol/L) | |  | 2.07 (2.00, 2.15) | 2.10 (2.01, 2.16) | 0.6 | 1.30 (0.91-2.11) | 1.24 (0.83-2.29) | 1.32 (0.86-2.57) |
| Albumin (g/L) | |  | 37.30 (34.65, 40.17) | 37.75 (36.40, 39.00) | 0.6 | 1.00 (0.74-1.37) | 0.96 (0.66-1.43) | 1.05 (0.72-1.60) |
| Sodium (mmol/L) | |  | 139 (138, 140) | 138 (137, 140) | 0.7 | 1.30 (0.93-1.94) | 1.34 (0.88-2.31) | 1.24 (0.84-2.03) |
| Folate (nmol/L) | |  | 25 (18, 30) | 21 (16, 26) | 0.2 | 0.76 (0.55-1.04) | 0.90 (0.60-1.32) | 0.65 (0.42-0.96) |
| Vitamin A (µmol/L) | | | 1.07 (0.89, 1.24) | 1.14 (0.85, 1.39) | 0.7 | **1.67 (1.21-2.37)** | **1.58 (1.07-2.41)** | **1.64 (1.13-2.45)** |
| Vitamin B_6_ vitamers: | |  |  |  |  |  |  |  |
| - PA (nmol/L) | |  | 26 (20, 36) | 25 (16, 34) | 0.6 | 0.96 (0.69-1.30) | 1.01 (0.66-1.43) | 0.91 (0.58-1.32) |
| - PLP (nmol/L) | |  | 28 (18, 30) | 24 (21, 30) | 0.8 | 0.85 (0.61-1.16) | 0.78 (0.49-1.16) | 0.93 (0.61-1.33) |
| - PL (nmol/L) | |  | 9.6 (7.5, 11.1) | 8.6 (6.7, 12.8) | 0.4 | 0.86 (0.59-1.17) | 0.96 (0.63-1.36) | 0.72 (0.40-1.14) |
| - PM (nmol/L) | |  | 0.20 (0.13, 0.30) | 0.20 (0.10, 0.20) | 0.3 | 0.91 (0.62-1.25) | 1.02 (0.66-1.44) | 0.75 (0.39-1.19) |
| - PN (nmol/L) | |  | 0.10 (0.10, 0.20) | 0.10 (0.00, 0.20) | >0.9 | 0.71 (0.49-1.00) | 0.74 (0.45-1.13) | 0.68 (0.42-1.04) |
| Vitamin B_12_ (pmol/L) | | | 324 (211, 400) | 417 (306, 662) | **0.025** | **0.68 (0.47-0.95)** | **0.46 (0.25-0.77)** | 0.87 (0.56-1.27) |
| Vitamin E (µmol/L) | | | 16.8 (14.0, 20.8) | 16.5 (13.1, 18.8) | 0.2 | **1.47 (1.07-2.07)** | **1.64 (1.11-2.52)** | 1.26 (0.86-1.87) |
| **Autoimmunity** | |  |  |  |  |  |  |  |
| LMOD1-IgG seropositivity | | | 20 (57%) | 17 (49%) | 0.5 | 1.39 (0.71-2.75) | 1.66 (0.73-3.84) | 1.17 (0.51-2.68) |
| **Inflammatory markers** | |  |  |  |  |  |  |  |
| AGP (g/L) | |  | 0.78 (0.67, 1.07) | 0.81 (0.68, 1.04) | 0.8 | 1.22 (0.90-1.66) | 1.23 (0.84-1.78) | 1.20 (0.80-1.76) |
| CRP (mg/L) | |  | 1 (1, 3) | 2 (1, 3) | 0.7 | 1.17 (0.86-1.63) | 1.21 (0.85-1.72) | 1.12 (0.67-1.71) |

**SUPPLEMENTARY FIGURES**


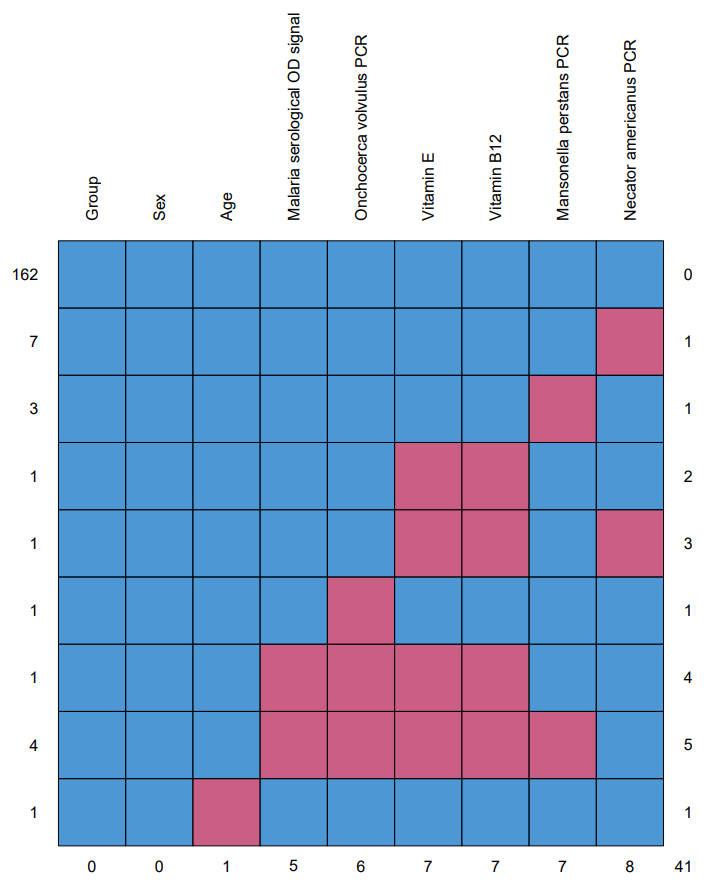


**Supplementary Figure 1** – Matrix of missing data. Blue: data available, red: data missing, columns: variables names (top) with number of total missing entries per variable (bottom), rows: unique patterns of missing data with number of missing variables for the respective pattern (right) and number of subjects with the respective pattern (left).


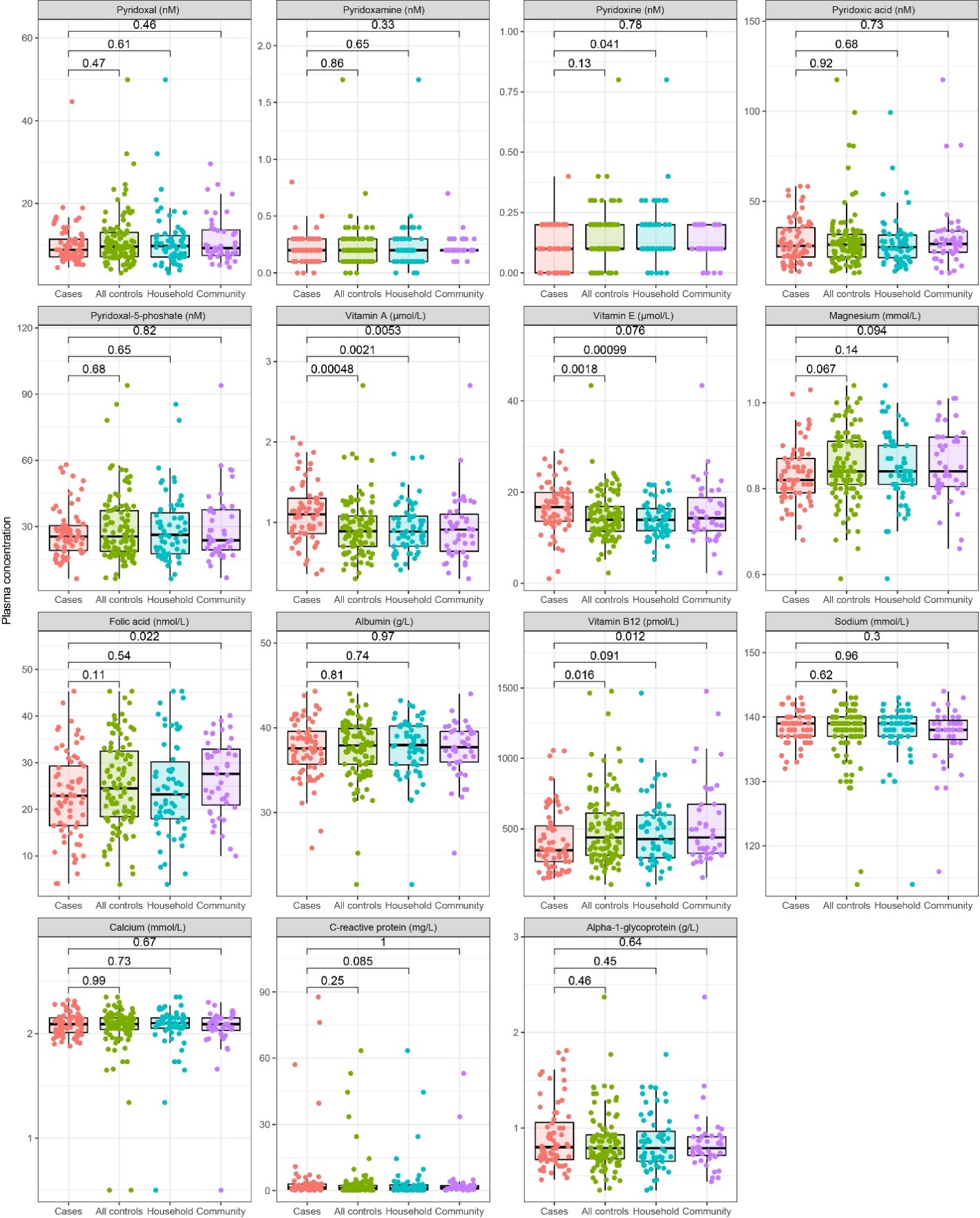


**Supplementary Figure 2** –Comparison of continuous nutritional markers between NS cases and household and community controls. The Wilcoxon signed-rank test was used for comparisons.


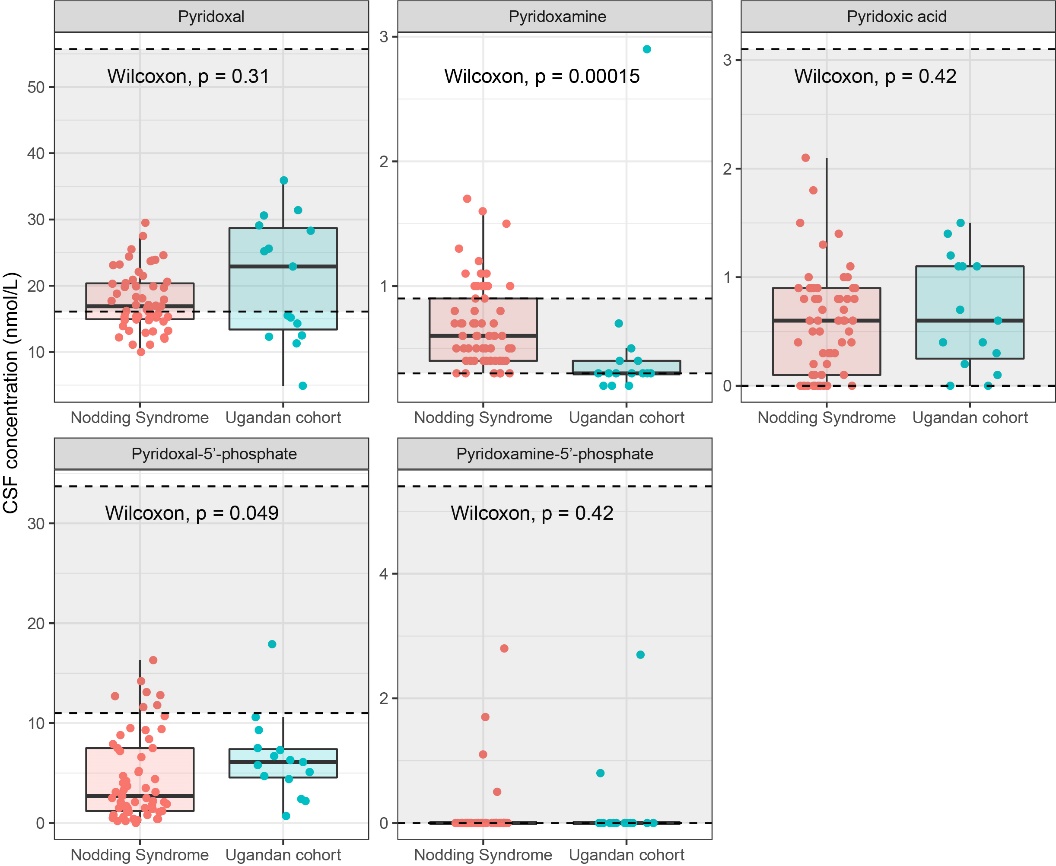


**Supplementary Figure 3** – Comparison of vitamin B_6_ vitamer concentrations in CSF from NS cases and Ugandan children with severe acute encephalopathy. Patients with quantifiable pyridoxine (PN) levels (n=3, all from the Ugandan cohort, suggestive of vitamin B_6_ supplementation) were excluded. The dark grey area indicates Dutch normal reference ranges for children 1-18 years of age (**Supplementary Table 2**). The Wilcoxon signed-rank test was used for comparisons.


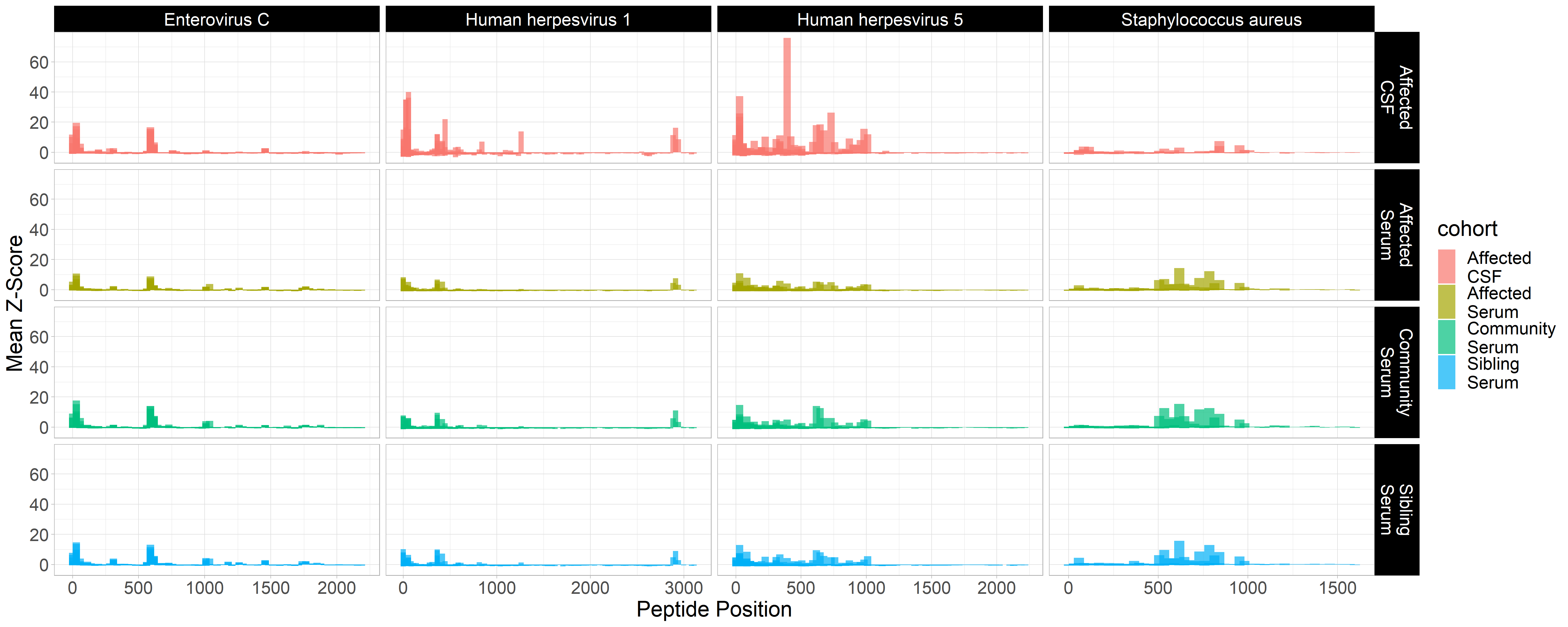

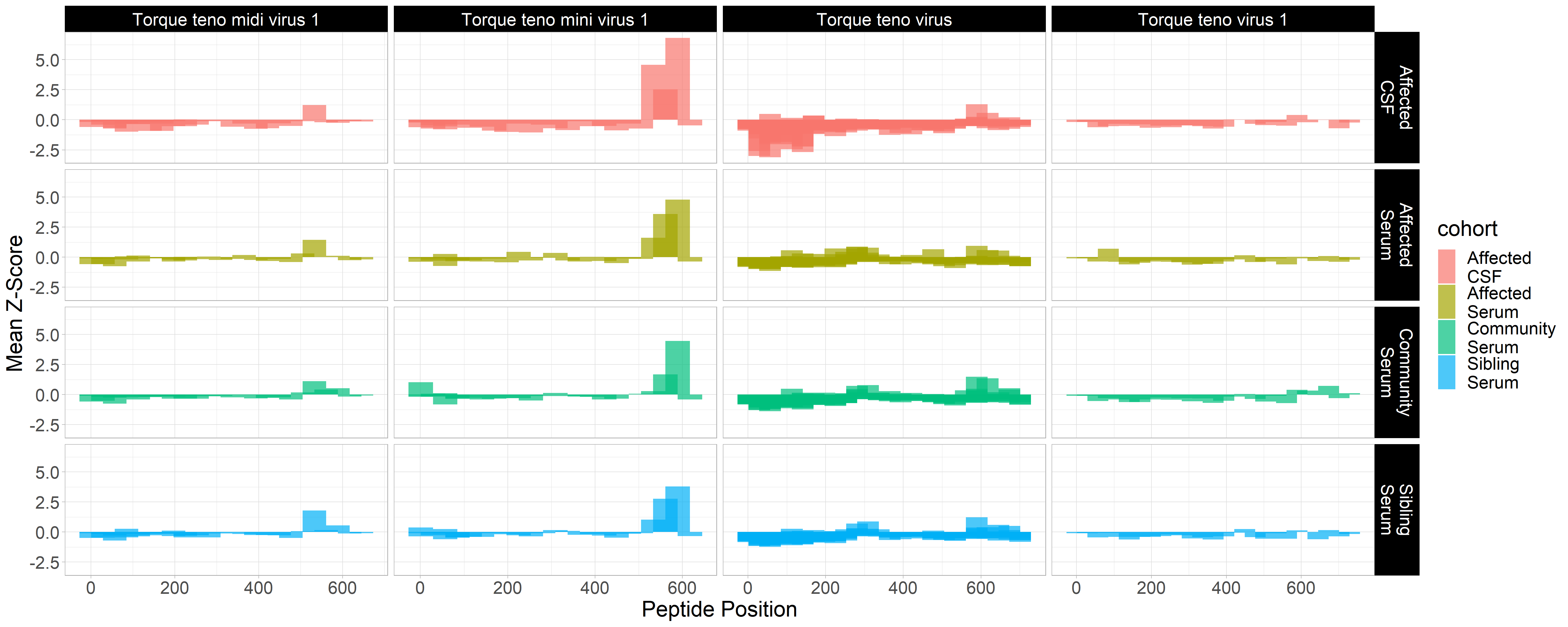

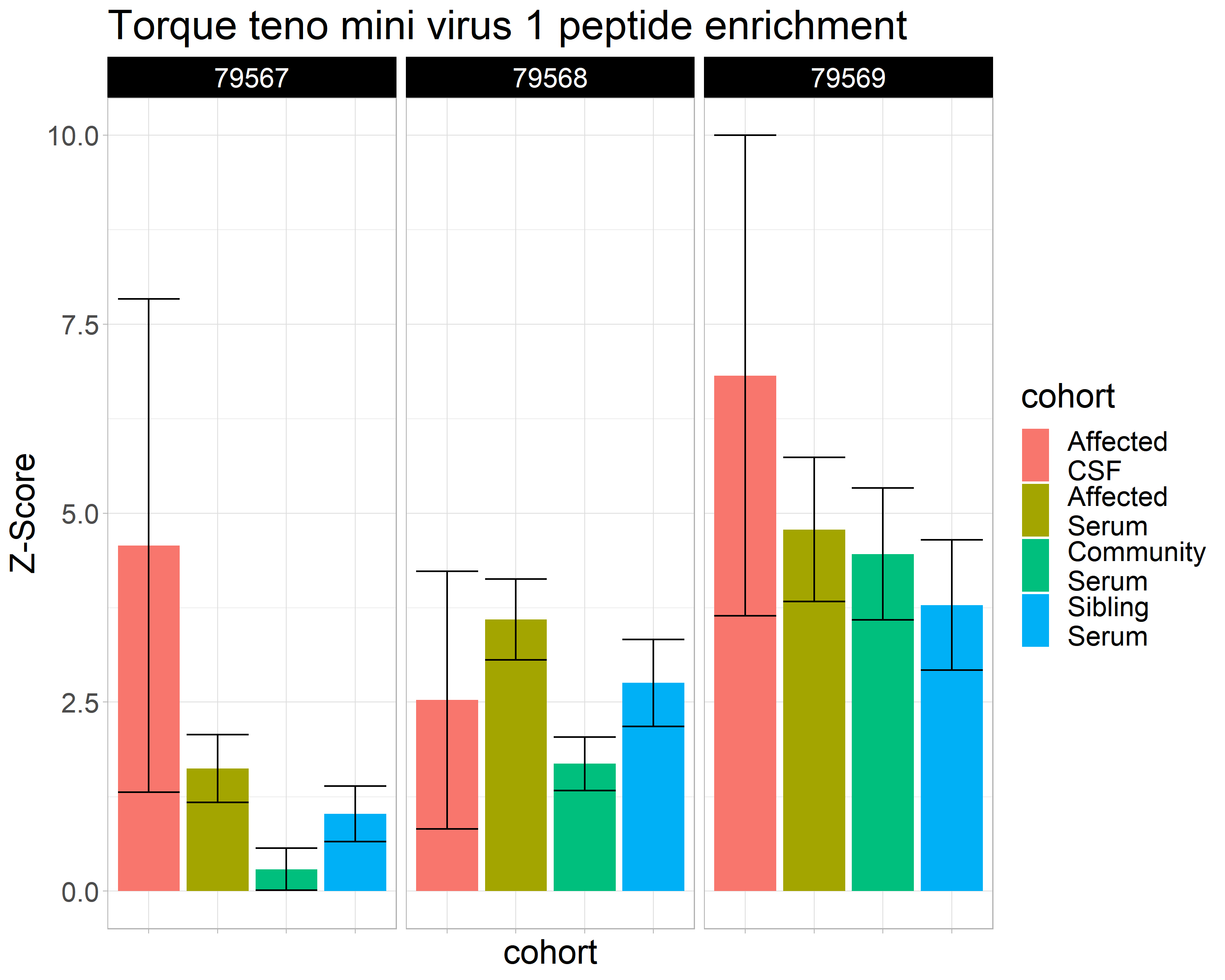


**A**

**B**

**C**

**Supplementary Figure 4** (**A**) Peptide enrichment score magnitude (Z-score) for all peptides available for four viruses. For each virus, average per-peptide z-score was calculated in each sample set. These average z-scores are plotted two viruses with CSF-enriched patterns: human herpesvirus 1 (HSV-1) and human herpesvirus 5 (CMV), and two pathogens without CSF-enrichment: enterovirus C and *Staphylococcus aureus*. (**B**) Peptide enrichment score magnitude (Z-score) for all peptides available from anelloviruses. (**C**) Peptide enrichment score magnitude (Z-score) for three TTMV1 peptides with relatively high CSF-enrichment.


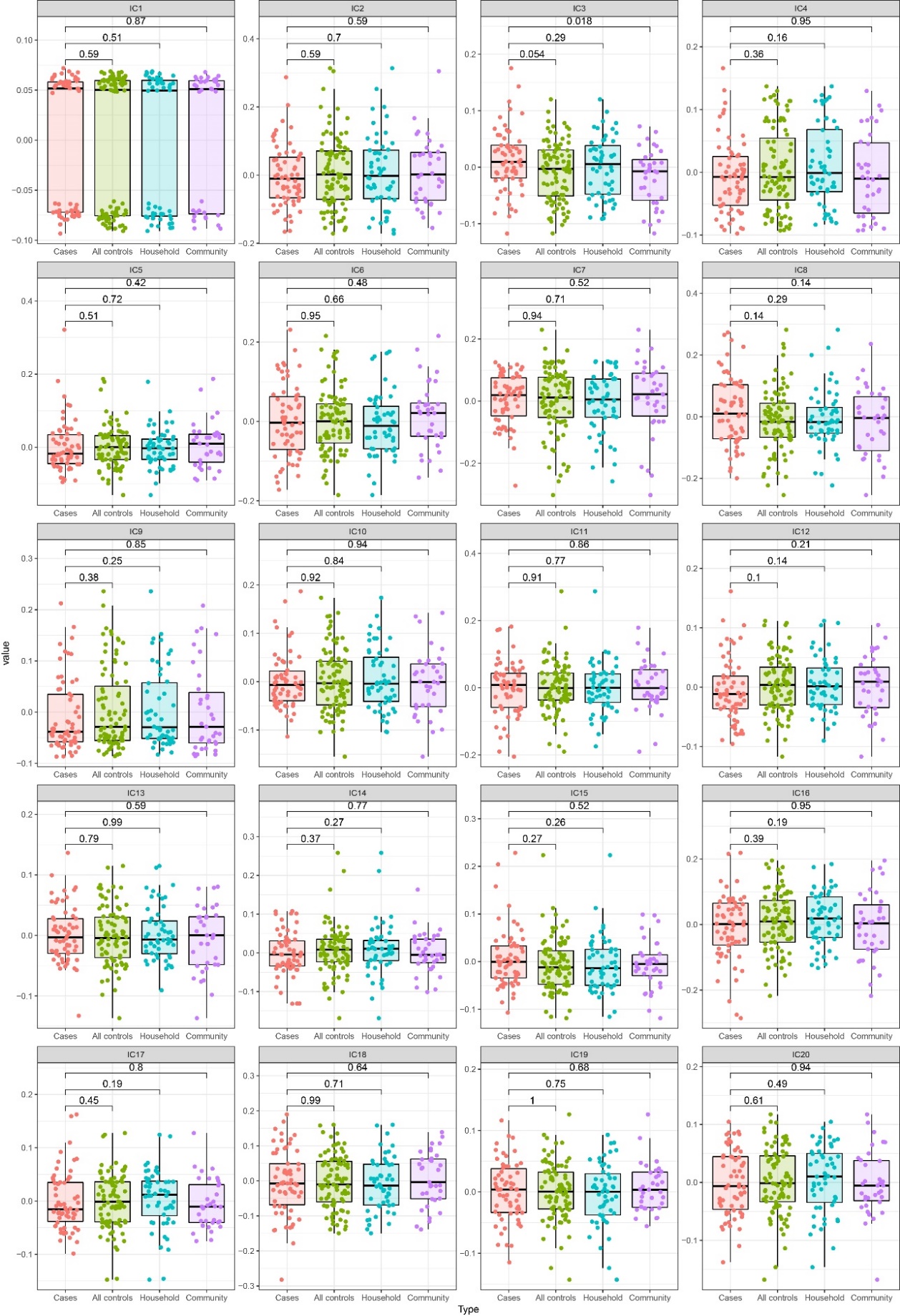


**Supplementary Figure 5** – Comparison of weights of 20 independent components identified in the whole blood gene expression dataset between NS cases and controls. The Wilcoxon signed-rank test was used for comparisons.


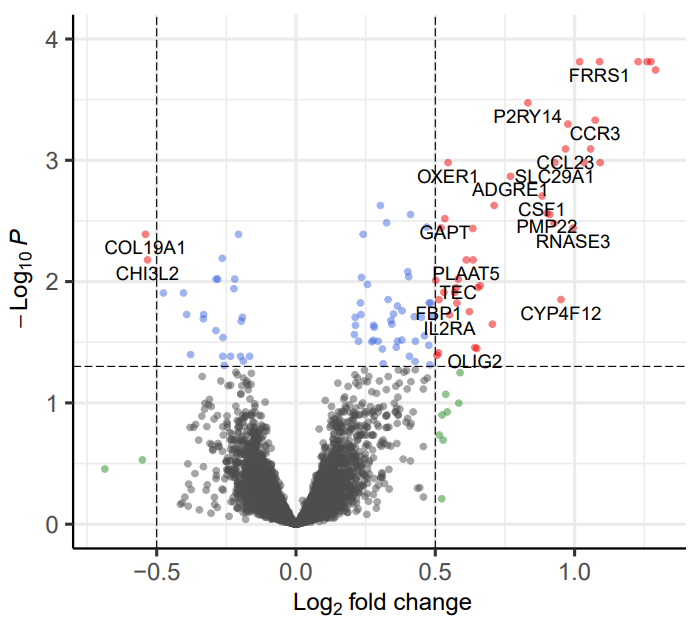


**Supplementary Figure 6 -** Volcano plot of patients seropositive versus seronegative for pan-filarial antibodies. Genes with an fold change >|2| (absolute value) and corrected p value <0.05 (t-test with false discovery rate correction) are highlighted in red and named.


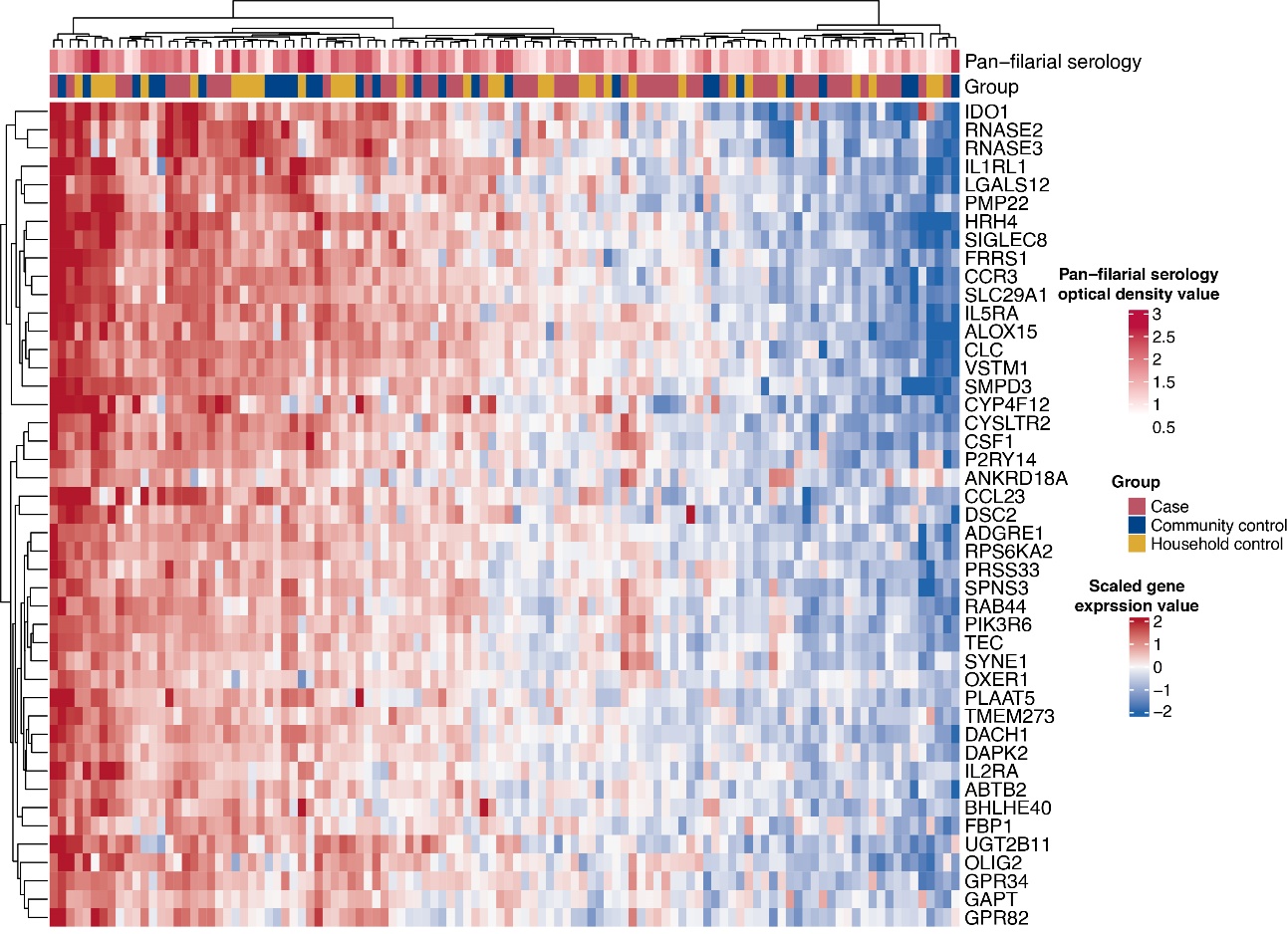


**Supplementary Figure 7** – Heatmap of expression levels of filarial-associated genes and study subjects, constructed with a complete agglomeration method (Heatmap function from the ComplexHeatmap R package version 2.10.0).

**
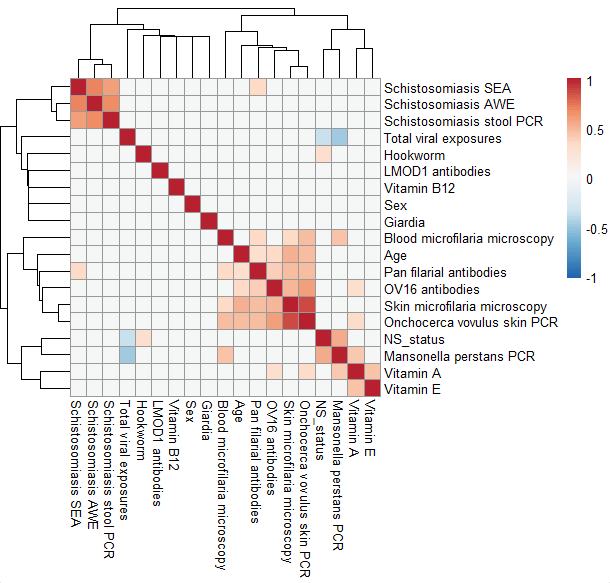
**

**Supplementary Figure 8** – Correlation matrix between dependent and independent variables. The colour of the squares indicate the correlation strength (r), only correlations with r>|0.3| (absolute value) are shown (cor_auto function from the qgraph R package version 1.9.4, which applies polychoric, polyserial or Pearson correlations based on the data type per variable).


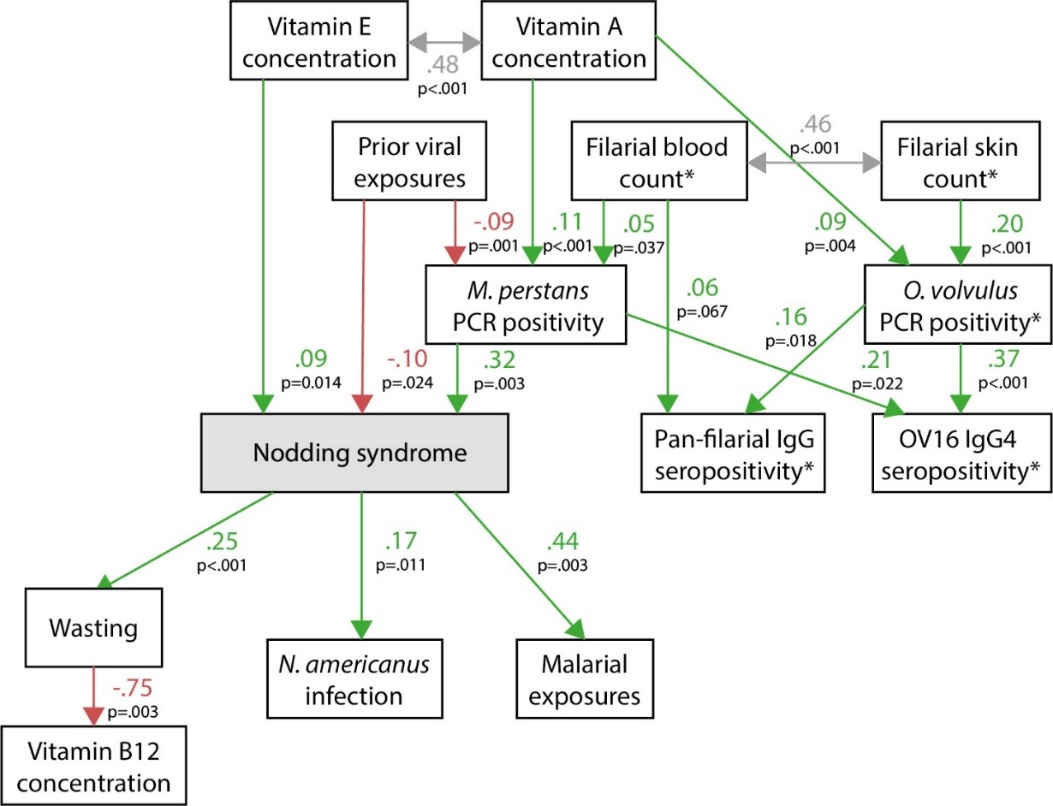


**Supplementary Figure 9** - Structural equation modelling of factors associated with nodding syndrome. Green arrows: positive associations, red arrows: negative associations, grey arrows: covariances, values: standardized coefficient estimates that indicate the relative strength of each association and respective p-values. The root mean square error of approximation (range 0 to 1, a smaller value indicates a better model fit) of the model was 0.045 (90% CI 0.020–0.065).


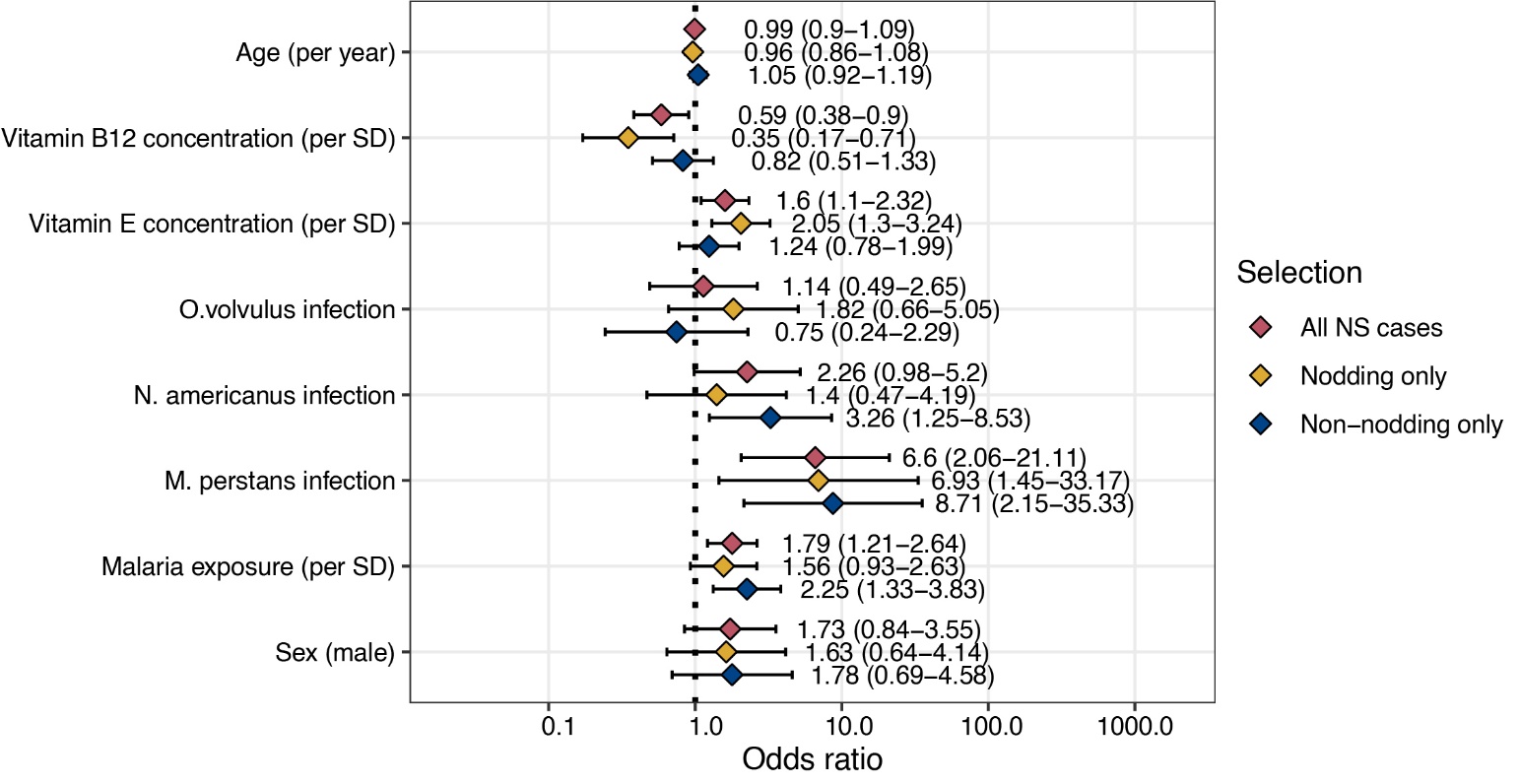


**Supplementary Figure 10 -** Multiple logistic regression of factors associated with disease comparing different subgroups of cases (all cases, only nodding cases, and only non-nodding cases).

**SUPPLEMENTARY REFERENCES**

1. Albersen M, Bosma M, Luykx JJ, et al. Vitamin B-6 vitamers in human plasma and cerebrospinal fluid. The American Journal of Clinical Nutrition [Internet] 2014 [cited 2021 Dec 29];100(2):587–92. Available from: https://academic.oup.com/ajcn/article/100/2/587/4576520
2. Haijes HA, van der Ham M, Gerrits J, et al. Direct-infusion based metabolomics unveils biochemical profiles of inborn errors of metabolism in cerebrospinal fluid. Mol Genet Metab [Internet] 2019 [cited 2021 Dec 29];127(1):51–7. Available from: https://pubmed.ncbi.nlm.nih.gov/30926434/
3. Haijes H, Willemsen M, van der Ham M, et al. Direct Infusion Based Metabolomics Identifies Metabolic Disease in Patients’ Dried Blood Spots and Plasma. Metabolites [Internet] 2019 [cited 2019 Oct 1];9(1):12. Available from: http://www.mdpi.com/2218-1989/9/1/12
4. Draijer LG, Froon-Torenstra D, van Weeghel M, et al. Lipidomics in Nonalcoholic Fatty Liver Disease: Exploring Serum Lipids as Biomarkers for Pediatric Nonalcoholic Fatty Liver Disease. J Pediatr Gastroenterol Nutr [Internet] 2020 [cited 2021 Dec 29];71(4):433–9. Available from: https://pubmed.ncbi.nlm.nih.gov/32947564/
5. Chong J, Wishart DS, Xia J. Using MetaboAnalyst 4.0 for Comprehensive and Integrative Metabolomics Data Analysis. Current Protocols in Bioinformatics [Internet] 2019 [cited 2021 Dec 29];68(1):e86. Available from: https://onlinelibrary.wiley.com/doi/full/10.1002/cpbi.86
6. Application of the enzyme-linked immunosorbent assay (ELISA) for the diagnosis of filariasis and echinococcosis - PubMed [Internet]. [cited 2022 Apr 27];Available from: https://pubmed.ncbi.nlm.nih.gov/7015635/
7. Niederhauser C, Tinguely C, Dreier J, et al. Comparison of a New IgG-EIA for the Detection of Anti-Plasmodium Antibodies with Two Currently Used Assays. Transfusion Medicine and Hemotherapy [Internet] 2021 [cited 2022 Apr 27];48(5):265–71. Available from: https://www.karger.com/Article/FullText/515842
8. Chernet A, Kling K, Sydow V, et al. Accuracy of Diagnostic Tests for Schistosoma mansoni Infection in Asymptomatic Eritrean Refugees: Serology and Point-of-Care Circulating Cathodic Antigen Against Stool Microscopy. Clin Infect Dis [Internet] 2017 [cited 2022 Apr 27];65(4):568–74. Available from: https://pubmed.ncbi.nlm.nih.gov/28430889/
9. Ampah KA, Nickel B, Asare P, et al. A Sero-epidemiological Approach to Explore Transmission of Mycobacterium ulcerans. PLOS Neglected Tropical Diseases [Internet] 2016 [cited 2022 Apr 27];10(1):e0004387. Available from: https://journals.plos.org/plosntds/article?id=10.1371/journal.pntd.0004387
10. Nickel B, Sayasone S, Vonghachack Y, Odermatt P, Marti H. Schistosoma mansoni antigen detects Schistosoma mekongi infection. Acta Trop [Internet] 2015 [cited 2022 Apr 27];141(Pt B):310–4. Available from: https://pubmed.ncbi.nlm.nih.gov/25116398/
11. Verweij JJ, Laeijendecker D, Brienen EAT, van Lieshout L, Polderman AM. Detection of Cyclospora cayetanensis in travellers returning from the tropics and subtropics using microscopy and real-time PCR. Int J Med Microbiol [Internet] 2003 [cited 2022 Apr 27];293(2–3):199–202. Available from: https://pubmed.ncbi.nlm.nih.gov/12868656/
12. Verweij JJ, Blangé RA, Templeton K, et al. Simultaneous detection of Entamoeba histolytica, Giardia lamblia, and Cryptosporidium parvum in fecal samples by using multiplex real-time PCR. J Clin Microbiol [Internet] 2004 [cited 2022 Apr 27];42(3):1220–3. Available from: https://pubmed.ncbi.nlm.nih.gov/15004079/
13. Praet N, Verweij JJ, Mwape KE, et al. Bayesian modelling to estimate the test characteristics of coprology, coproantigen ELISA and a novel real-time PCR for the diagnosis of taeniasis. Trop Med Int Health [Internet] 2013 [cited 2022 Apr 27];18(5):608–14. Available from: https://pubmed.ncbi.nlm.nih.gov/23464616/
14. Meurs L, Polderman AM, Vinkeles Melchers NVS, et al. Diagnosing Polyparasitism in a High-Prevalence Setting in Beira, Mozambique: Detection of Intestinal Parasites in Fecal Samples by Microscopy and Real-Time PCR. PLOS Neglected Tropical Diseases [Internet] 2017 [cited 2022 Apr 27];11(1):e0005310. Available from: https://journals.plos.org/plosntds/article?id=10.1371/journal.pntd.0005310
15. Cools P, Vlaminck J, Albonico M, et al. Diagnostic performance of a single and duplicate Kato-Katz, Mini-FLOTAC, FECPAKG2 and qPCR for the detection and quantification of soil-transmitted helminths in three endemic countries. PLOS Neglected Tropical Diseases [Internet] 2019 [cited 2022 Apr 27];13(8):e0007446. Available from: https://journals.plos.org/plosntds/article?id=10.1371/journal.pntd.0007446
16. Verweij JJ. Validation and maintaining laboratory developed molecular tests compliant with ISO15189 for diagnosis of intestinal parasitic infections. Expert Rev Mol Diagn [Internet] 2021 [cited 2022 Apr 27];Available from: https://pubmed.ncbi.nlm.nih.gov/34424112/
17. Mina MJ, Kula T, Leng Y, et al. Measles virus infection diminishes preexisting antibodies that offer protection from other pathogens. Science (1979) [Internet] 2019 [cited 2020 Sep 10];366(6465):599–606. Available from: http://science.sciencemag.org/
18. Xu GJ, Kula T, Xu Q, et al. Comprehensive serological profiling of human populations using a synthetic human virome. Science (1979) [Internet] 2015 [cited 2022 Jan 4];348(6239). Available from: https://www.science.org/doi/abs/10.1126/science.aaa0698
19. vander Ham M, Albersen M, de Koning TJ, et al. Quantification of vitamin B6 vitamers in human cerebrospinal fluid by ultra performance liquid chromatography-tandem mass spectrometry. Anal Chim Acta [Internet] 2012 [cited 2022 Apr 27];712:108–14. Available from: https://pubmed.ncbi.nlm.nih.gov/22177072/
20. Spencer PS, Mazumder R, Palmer VS, et al. Environmental, dietary and case-control study of Nodding Syndrome in Uganda: A post-measles brain disorder triggered by malnutrition? J Neurol Sci. 2016;369:191-203. doi:10.1016/J.JNS.2016.08.023
21. Angues RV, Palmer VS, Mazumder R, Okot C, Spencer PS. Preliminary seroprevalence study of neurotropic virus antibodies in Nodding syndrome. eNeurologicalSci. 2022;29:100423. doi:10.1016/J.ENSCI.2022.100423
22. Shrock EL, Timms RT, Kula T, et al. Germline-encoded amino acid-binding motifs drive immunodominant public antibody responses. Science. 2023;380(6640):eadc9498. doi:10.1126/SCIENCE.ADC9498
23. Hotterbeekx A, Raimon S, Abd-Elfarag G, et al. Onchocerca volvulus is not detected in the cerebrospinal fluid of persons with onchocerciasis-associated epilepsy. International Journal of Infectious Diseases 2020;91:119–23.
24. Colebunders R, Mandro M, Mokili JL, et al. Risk factors for epilepsy in Bas-Uélé Province, Democratic Republic of the Congo: a case–control study. International Journal of Infectious Diseases 2016;49:1–8.
25. Rishniw M, Barr SC, Simpson KW, Frongillo MF, Franz M, Dominguez Alpizar JL. Discrimination between six species of canine microfilariae by a single polymerase chain reaction. Vet Parasitol [Internet] 2006 [cited 2022 Jan 8];135(3–4):303–14. Available from: https://pubmed.ncbi.nlm.nih.gov/16289566/
26. Jiménez M, González LM, Carranza C, et al. Detection and discrimination of Loa loa, Mansonella perstans and Wuchereria bancrofti by PCR–RFLP and nested-PCR of ribosomal DNA ITS1 region. Experimental Parasitology 2011;127(1):282–6.
