# Supplementary material for: Parasitic, bacterial, viral, immune-mediated, metabolic and nutritional factors associated with nodding syndrome
Source: Brain Commun. 2023 Aug 17;5(5):fcad223. doi: 10.1093/braincomms/fcad223 (PMC10507744; doi:10.1093/braincomms/fcad223)
Supplement: fcad223_Supplementary_Data [file fcad223_supplementary_data.zip › Supplementary_Materials_Statistical_analysis_plan.docx]

**Statistical analysis plan**

Because several analyses were added to the original study protocol, this statistical analysis plan was written after the data collection was completed. For details on the study background and methodology, we refer to the study protocol.

**List of additional analyses added to the original study protocol**:

- Viral serological profiling (VirScan)
- Metabolomics on plasma and cerebrospinal fluid
- Lipidomics on plasma
- Quantification of all B_6_ vitamers in plasma and cerebrospinal fluid
- Serological screening for various parasites
- Extended screening for multiple nutrient markers in plasma
- Testing for potential antineuronal antibodies in cerebrospinal fluid and plasma using rat-brain immunohistochemistry

**Primary study aim**

To explore nutritional, parasitic, viral, metabolic, immune-mediated risk factors for NS.

**Secondary study aim**

To construct a hypothetical causal model based on identified risk factors using structural equation modelling.

**Reporting of exposure variables**

All exposure variables will be presented using descriptive statistics: mean and standard deviation for normally distributed continuous data, median and interquartile range for non-normally distributed data, and county and percentages for dichotomous data.

**Correction for multiple testing**

False discovery rate testing will be applied to the results of metabolomics, lipidomics, viral serological profiling, and gene expression profiling.

**Analyses**

Diagnostic-assay specific statistical methodology is supplied in the **(Supplementary) Methods** section.

Primary study aim

The association between exposure variables and nodding syndrome will initially be explored by conditional and conventional logistic regression using R (version 4.0.3). All variables with a p-value <0.05 and those deemed clinically relevant (e.g. *Onchocerca volvulus* infection, based on results from prior studies) will subsequently combined by multiple logistic regression analysis (MLRA). A stringent p-value cut-off is chosen because of the large number of predictors relative to the sample size. Results of regression analyses will be reported as odds ratios with 95% confidence intervals.

Secondary study aim

A hypothetical causal model will be based on the results of the simple and multiple regression analysis, biological knowledge of associations, and hypothetical plausibility of novel associations in AMOS (version 14). Associations between variables will be retained or removed based on the effect on the root mean square error of approximation (RMSEA, e.g. if a new association considerably increased the RMSEA it will be removed).

**Missing data**

For simple (conditional and conventional) logistic regression, we will use complete observations only. Multiple logistic regression will be performed with missing data and with missing data imputation, assuming missing data at random with 5 iterations using all variables included in the MLRA using MICE R package (version 3.13.0), based on the pattern of missing data (see **Supplementary Methods** and **Supplementary Figure 1**). Missing data in structural equation modelling will be estimated using full information maximum likelihood by AMOS (version v14).

**Controlling for measured and unmeasured confounders**

The multiple logistic regression model and structural equation model will be corrected for age and sex, as both are known to be associated with Nodding syndrome and several studied risk factors (e.g. filarial infections and nutrient levels).

Furthermore, we specifically hypothesized if ivermectin could be a confounder in the association between *Onchocerca volvulus* infection and Nodding syndrome (**Figure 1**). For this to be the case, ivermectin would directly modify the risk of developing Nodding syndrome, independent of its effect on *O. volvulus*, which we estimate to be unlikely. We therefore did not consider ivermectin to be a possible confounder

***O. volvulus* infection**

**Nodding syndrome**

**Ivermectin use**

Studied association

Known association (ivermectin clears microfilaria)

Ivermectin causes Nodding syndrome

**Figure 1** – Possible hypothesis how ivermectin use could confound the association between *O. volvulus* infection and Nodding syndrome
